# No common denominator for breast cancer lymph node metastasis

**DOI:** 10.1038/sj.bjc.6602794

**Published:** 2005-09-27

**Authors:** B Weigelt, L F A Wessels, A J Bosma, A M Glas, D S A Nuyten, Y D He, H Dai, J L Peterse, L J van't Veer

**Affiliations:** 1 Division of Experimental Therapy, The Netherlands Cancer Institute, 1066 CX Amsterdam, The Netherlands; 2 Division of Diagnostic Oncology, The Netherlands Cancer Institute, 1066 CX Amsterdam, The Netherlands; 3 Information and Communication Theory Group, Delft University of Technology, 2600 GA Delft, The Netherlands; 4 Division of Radiotherapy, The Netherlands Cancer Institute, 1066 CX Amsterdam, The Netherlands; 5 Rosetta Inpharmatics LLC, Seattle, WA 98109, USA

**Keywords:** breast cancer, lymph node metastasis, expression profiling, prognosis marker, CXCR4, VEGF

## Abstract

The axillary lymph node status is the most powerful prognostic factor for breast cancer patients to date. The molecular mechanisms that control lymph node metastasis, however, remain poorly understood. To define patterns of genes or gene regulatory pathways that drive breast cancer lymph node metastasis, we compared the gene expression profiles of 15 primary breast carcinomas and their matching lymph node metastases using microarrays. In general, primary breast carcinomas and lymph node metastases do not differ at the transcriptional level by a common subset of genes. No classifier or single gene discriminating the group of primary tumours from those of the lymph node metastases could be identified. Also, in a series of 295 breast tumours, no classifier predicting lymph node metastasis could be developed. However, subtle differences in the expression of genes involved in extracellular-matrix organisation and growth factor signalling are detected in individual pairs of matching primary and metastatic tumours. Surprisingly, however, different sets of these genes are either up- or downregulated in lymph node metastases. Our data suggest that breast carcinomas do not use a shared gene set to accomplish lymph node metastasis.

Distant metastases are the main cause of death in breast cancer patients. To successfully establish a metastatic colony, primary tumour cells have to invade their surrounding host tissue and enter the bloodstream. Subsequently, the neoplastic cells must survive in the blood circulation, arrest in capillary beds of distant organs and extravasate into the parenchyma. Finally, tumour cells need to proliferate and establish vascularisation (Fidler, 1978; Chambers *et al*, 2002). The biology of this multistep metastatic process has mainly been studied for tumour cells that disseminate via the haematogenous route. In breast cancer, however, the axillary lymph nodes are often the first sites to harbour metastases (Stacker *et al*, 2002). These regional metastases are not life threatening *per se*, yet their presence or absence is the most powerful prognostic factor for disease course that is currently available for breast cancer patients (McGuire, 1987; Foster, 1996). Approximately one-third of women with breast cancer and tumour-negative lymph nodes develop distant metastases, whereas about one-third of patients with positive lymph nodes remain free of distant metastases 10 years after local therapy (Rosen *et al*, 1989; Hellman, 1994). Given this lack of correlation between the lymph node status and tumour recurrence at distant organs, it remains unclear whether metastasis to distant sites proceeds sequentially from lymph node metastasis or in parallel by a haematogenous route (Chambers *et al*, 2002). Moreover, it is still under debate to what extent lymph node metastasis depends on lymph vessel growth or on invasion of existing lymph vessels (Padera *et al*, 2002; Nathanson, 2003; Williams *et al*, 2003). The identification of molecules promoting lymphangiogenesis and lymphatic metastasis in mouse models, such as the vascular endothelial growth factor (VEGF) family members C and D, suggests that lymph vessel neogenesis is an essential step in the process of lymph node metastasis (Karpanen *et al*, 2001; Mandriota *et al*, 2001; Skobe *et al*, 2001; Stacker *et al*, 2001). The invasion into the lymph nodes has also been suggested to be activated by chemokines, including CXCL12 that acts on its receptor CXCR4 (Muller *et al*, 2001). Furthermore, lymph node metastasis has been proposed to be a passive, mechanical process, based on the fluid pressure within a tumour, washing cells into draining lymphatics (Hartveit, 1990). However, once passively transported cells have reached the lymph nodes, they have to be able to proliferate in this new environment in order to form a metastasis.

Thus, there is a need for a better understanding of the molecular basis of breast cancer initiation and metastasis to improve prognosis prediction and develop targeted, molecular-based therapies. In the present study, we compared the gene expression profiles of primary tumours and their matching lymph node metastases obtained from the same patient. Our aim was to define patterns of genes or gene regulatory pathways that drive the metastatic dissemination of primary breast cancer cells to the lymph nodes.

## MATERIALS AND METHODS

### Tissue samples

A total of 15 breast cancer patients with lymph node metastases at diagnosis, four patients with two primary breast carcinomas and a metastasis, and additional primary tumour samples (*n*=31) for real-time PCR analysis were selected from the fresh-frozen tissue bank of the Netherlands Cancer Institute. The tumour and metastatic material was snap-frozen in liquid nitrogen within 1 h after surgery. Before and after cutting sections for RNA isolation, one slide was stained with haematoxylin and eosin to select only samples of 60% or more tumour cells in primary tumours and of 70% or more in lymph node metastases. Patients had no prior malignancies. A tumour was oestrogen receptor-*α* (ER-*α*) negative when less than 10% of the cells showed staining by immunohistochemistry.

For real-time PCR analysis, fresh-frozen material from normal lymph nodes (*n*=10) and normal skin (*n*=10) was obtained from patients without breast cancer undergoing a preventive breast ablation and normal breast tissue (*n*=10) was obtained from healthy women undergoing breast reduction. Additionally, total RNA of normal bone marrow, normal liver and normal lung was obtained from BD Biosciences (Palo Alto, USA).

This study was approved by the Medical Ethical Committee of the Netherlands Cancer Institute.

### RNA isolation and amplification, cRNA labelling and hybridisation

RNA isolation and amplification were performed as described previously (Weigelt *et al*, 2003). Amplification yields were 1000–2000-fold and quality was checked on agarose gel. Detailed protocols for RNA isolation and amplification can be found at http://www.nki.nl/nkidep/pa/microarray/protocols.htm.

cRNA labelling and hybridisation were performed as described previously (Weigelt *et al*, 2003). The reference pool consisted of pooled cRNA of equal amounts of 100 primary breast tumours. For each tumour and metastasis, two hybridisations were performed using a reversal fluorescent dye. Detailed protocols for cRNA labelling and hybridisation can be found at http://www.nki.nl/nkidep/pa/microarray/protocols.htm.

Fluorescent images of the microarrays were obtained using the Agilent DNA microarray scanner (Agilent Technologies, Palo Alto, USA). Fluorescent intensities of the images were quantified using ImaGene 5 (Biodiscovery, Marina Del Rey, USA) and corrected for background noise. The original data are available at http://www.nki.nl/nkidep/pa/microarray.

### Microarray slides

Complementary DNA (cDNA) microarray slides were manufactured at the Central Microarray Facility (CMF) of the Netherlands Cancer Institute, Amsterdam, The Netherlands. Sequence-verified cDNA clones (InVitrogen, Huntsville, USA) were spotted using the Microgrid II arrayer (Apogent, Cambridgeshire, UK) with a complexity of 19 200 spots per glass slide. The complete list of genes and controls spotted on the cDNA arrays, as well as detailed protocols for spotting and preparation of the slides, is available on the CMF website (http://microarrays.nki.nl/download/geneid.html, http://microarrays.nki.nl/download/protocols.html).

### Analysis and statistics

Fluorescence intensities of scanned images were quantified, normalised and ratios were calculated and compared to the intensities of the reference pool (Yang *et al*, 2002). Weighted averages and confidence levels were computed according to the Rosetta Error Model (Hughes *et al*, 2000). To determine genes that discriminate between primary tumours and metastases, we employed a supervised classification method using a nearest prototype classifier, and a leave-one-out crossvalidation method (van 't Veer *et al*, 2002).

A ‘predicting analysis of microarrays’ (PAM) was performed to find genes that accurately predict classes based on class labels (supervised analysis) (Tibshirani *et al*, 2002), using all 18 336 genes of the array. After training, a 10-fold balanced crossvalidation was employed.

Differentially expressed genes between primary tumours and lymph node metastases were selected by the ‘significance analysis of microarrays’ (SAM) (http://www-stat.stanford.edu/~tibs/SAM) (Tusher *et al*, 2001). The input criteria selected for SAM included a Delta of 0.4 and one-fold or greater expression in the primary breast tumour group as compared to the lymph node metastases group using all 18 336 genes. In addition, a paired two-class SAM analysis was performed to identify genes consistently regulated between primary and metastatic tumour pairs, using all genes.

Gene clustering and tumour clustering were performed as described previously (Weigelt *et al*, 2003). For tumour clustering, complete linkage clustering was based on Xdev (defined as log(ratio) divided by error of log(ratio)) values across all 18k genes. Mapping by multidimensional scaling was performed as described previously (Weigelt *et al*, 2003). The permutation test to compute the within-pair–between-pair scatter ratio (WPBPSR) was repeated 20 000 times.

### Additional microarray information

The description of this study followed the MIAME guidelines issued by the Microarray Gene Expression Data Group (Brazma *et al*, 2001).

### Real-time quantitative PCR

A 1 *μ*g portion of total RNA was used for cDNA synthesis, as described previously (Lambrechts *et al*, 1999). Real-time quantitative PCR primers (Sigma Genosys, Cambridge, UK) and eventually 5′-fluorescently FAM labelled probes (Applied Biosystems, Nieuwerkerk a/d IJssel, The Netherlands) for matrix metalloprotease (MMP)3 and MMP9 were selected using the Perkin Elmer Primer Express® software (PE, Foster City, USA). The primer and probe sequences of VEGF-C, VEGF-D, CXCR4 and CXCL12 were selected from the literature (Niki *et al*, 2000; Schrader *et al*, 2002; Van Trappen *et al*, 2003) (Supplementary Table S1). Commercially available primers and probes for GAPDH and *β*-actin were used (Applied Biosystems) as housekeeping genes. The quantities found for the *β*-actin control and marker gene in singleplex reactions (ABI PRISM 7700, Applied Biosystems) were used to calculate the relative quantity of gene expression and that of GAPDH to confirm *β*-actin expression. Each experiment was performed in triplicate. The quality control of the PCR reactions was assessed by standardised PCR conditions, including in each experiment a genomic DNA control and a negative nontemplate control.

## RESULTS

### Gene expression profiling of primary breast carcinomas and matching lymph node metastases

We selected 15 breast cancer patients with axillary lymph node metastases at diagnosis whose invasive primary and metastatic tumours were stored in the tissue bank of the Netherlands Cancer Institute. No other selection criteria regarding age of the patient, ER status, tumour diameter or histological type of breast carcinoma were applied (Table 1). The patients had no prior malignancies and did not receive neo-adjuvant treatment. At the most recent follow-up (median 2.7 years), four patients (patient number 7, 8, 11 and 14) developed distant metastases.

We used human 18k cDNA microarrays to study the gene expression profiles of matching primary breast tumours and lymph node metastases and to gain an insight into specific changes associated with breast cancer metastasis to the lymph nodes. First, we employed a supervised classification method to identify genes that could discriminate the group of primary tumours from that of lymph metastases. The top ranked genes to separate the two classes in a nearest prototype classifier were determined and used in a crossvalidation procedure (Hughes *et al*, 2000; van 't Veer *et al*, 2002). No classifier, employing an incremental number of genes, which performed significantly better than random classification could be determined (data not shown). A second supervised analysis, the PAM, was used to classify and predict the category of the primary tumours and lymph node metastases on the basis of their gene expression profiles (Tibshirani *et al*, 2002). No subset of genes could be identified using PAM that can distinguish primary from metastatic tumours since the classification accuracy obtained from the crossvalidation procedure never exceeded 57% (Supplementary Figure S1). We further used the SAM (Tusher *et al*, 2001) to select genes differentially expressed between the primary breast carcinomas and the lymph node metastases. The SAM did not identify a single gene that is differentially expressed between the two groups (Supplementary Figure S2). Also a paired two-class SAM analysis did not find significant genes that are consistently regulated between primary and metastastic tumours (false discovery rate (FDR) of 5%) (data not shown). When we lowered the FDR to 10%, 14 genes were identified to be uniformly regulated between primary tumours and lymph node metastases (data not shown). This set of genes is, however, a very small subset from the 18 336 genes analysed with a relatively high FDR. In summary, our findings suggest that the primary breast carcinomas and lymph node metastases do not differ at the transcriptional level by a common subset of genes.

**Figure 1:**
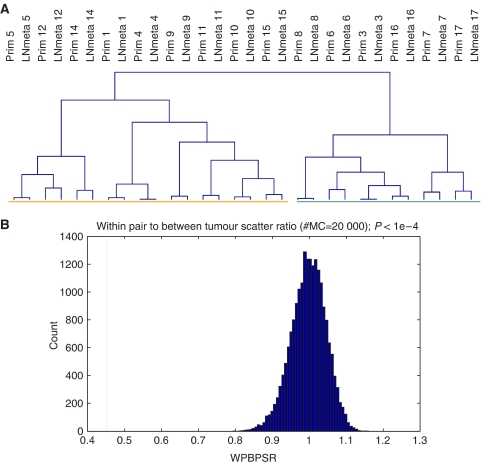
(**A**) Unsupervised hierarchical clustering of 30 primary breast carcinomas and lymph node metastases from 15 patients, measured over 18 336 genes. The dendrogram has two large branches; the orange bar represents ER-*α*-negative and the green bar ER-*α*-positive tumours. Alignment of all matching pairs was established. (**B**) Permutation test of the WPBPSR. Blue: null hypothesis distribution. Distribution after randomisation of the labels of the primary and metastatic tumours, repeated 20 000 times (WPBPSR=1±0.05). The red line represents the WPBPSR of the 15 matching pairs (WPBPSR=0.45; *P*<0.0001). Prim=primary tumour; LNmeta=lymph node metastasis; Prim *n*, LNmeta *n* (*n*=1–17)=patient number primary tumour, patient number lymph node metastasis, respectively.

To further scrutinise our results, we examined the similarity between primary and matching metastatic tumours. Unsupervised hierarchical clustering, the grouping of tumours based on their similarity measured overall genes on the array, revealed that the gene expression profiles of primary breast and matching regional metastatic tumours are highly alike (Figure 1A). The division of the dendrogram into two branches is based on the highly dominant ER-*α* expression profile displayed by nine of the 15 tumours and matching metastases (Gruvberger *et al*, 2001; van 't Veer *et al*, 2002; Weigelt *et al*, 2003).

A multidimensional scaling analysis further emphasises the high similarity in overall gene expression between primary breast carcinomas and their lymph node metastases, since all matching primary and metastatic tumours, except those of patient 6, established a pair (Supplementary Figure S3).

Given the relatively small number of samples included in this study, it is essential to ascertain that the similarity we observed between primary and metastatic tumours was not a result of chance. Therefore, a computational analysis was performed to establish the WPBPSR (Weigelt *et al*, 2003). Subsequently, we determined the statistical significance of this WPBPSR for the 15 given pairs by a permutation test. The similarity between matching pairs of primary breast carcinomas and lymph node metastases was shown to be significantly higher than the similarity between random pairs (WPBPSR 0.45 *vs* 1.0±0.05; *P*<0.0001) (Figure 1B). This finding demonstrates that the similarity within the matching pairs was not due to chance, but rather that the expression profiles of primary breast carcinomas are highly similar to their corresponding metastatic lesions.

To validate our finding that gene expression profiles of primary breast carcinomas are maintained in their lymph node metastases, a random subset of samples from our matching pairs (pair number 3, 4, 5, 12, 16, 17) was re-profiled and analysed using a different platform of inkjet-synthesised oligonucleotide microarrays, containing approximately 25 000 human genes. The primary tumours were not hybridised against a reference pool, but directly against their matching lymph node metastases obtained from the same patient. Using different analytical approaches, including parametric and nonparametric methods, no significant universal differences between the groups of primary and metastatic tumours could be found (data not shown). Nonetheless, an unaccordant difference for individual pairs was observed in a small number of genes comparable to false discovery.

**Figure 2:**
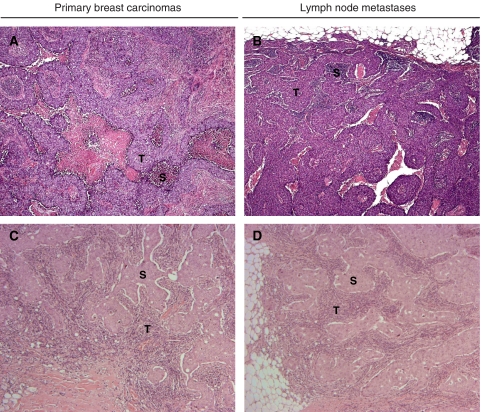
Haematoxylin and eosin staining of two paraffin-embedded primary infiltrative ductal breast carcinomas and their matching lymph node metastases (× 5). (**A, C**) Normal mammary gland tissue next to tumour cells. (**B, D**) The lymph node capsule adjacent to tumour cells. S=stromal cells; T=tumour cells.

The similarity in gene expression detected between primary tumours and their affiliated lymph node metastases is also reflected in the similarity of their histology (Figure 2). Although the morphological spectrum of breast cancers varies widely, the resemblance of the phenotypes of the pairs of primary tumours and lymph node metastases is striking. Metastases in the lymph nodes (Figure 2B and D) share distinct histological characteristics, like the growth pattern, with their primary ductal carcinomas (Figure 2A and C). Phenotypically, primary tumour and metastasis are visually distinguishable only by the normal mammary gland tissue and the lymph node capsule adjacent to the tumour mass itself.

### Similarity of primary breast carcinomas and matching metastases based on tumour-specific genes

**Figure 3:**
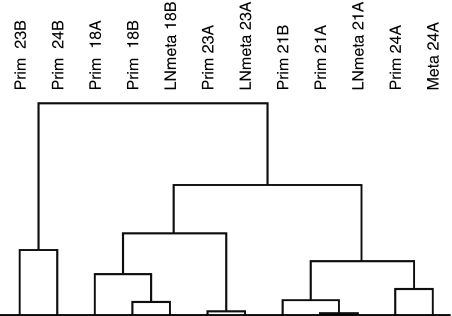
Unsupervised hierarchical clustering of eight primary breast carcinomas, obtained from four patients with two primary tumours, and matching metastases, measured over 18 336 genes. Alignment of primary tumours with their metastases, not with the second primary tumour, is shown. Prim=primary tumour; LNmeta=lymph node metastasis; Meta=distant metastasis; Prim *n*, LNmeta/Meta *n* (*n*=18, 21, 23, 24)=patient number primary tumour, patient number metastasis, respectively; Prim *n*A, Prim *n*B=two primary tumours; LN*n*A/B=lymph node metastasis developed from primary tumour A or B.

Since both primary and metastatic tumour tissues were derived from one individual, we attempted to show that the similarity in overall gene expression between primary breast carcinomas and their metastases is based on genes specific for the primary tumour rather than specific for the patient. We selected two patients who developed bilateral breast cancer and a lymph node metastasis of either one of the two primary tumours (patient 18 and 21), one patient with contralateral breast cancer and a distant metastasis in the ovary (patient 24) and one patient who developed two primary breast carcinomas in one breast and a lymph node metastasis (patient 23) (for patient and tumour characterisation, see Table 2). The primary and metastatic tumours were then analysed for their gene expression profiles. Unsupervised hierarchical clustering using all 18 366 genes underscored our histological observations, namely that the gene expression profile of a primary breast tumour is more similar to that of its affiliated metastasis than to that of the second primary tumour (Figure 3).

### Genes differentially expressed between pairs of primary breast tumours and matching lymph node metastases

To gain an insight into the pattern of genes or gene regulatory pathways allowing the primary tumours to metastasise to the lymph nodes, we selected genes that were significantly expressed in both primary tumour and lymph node metastasis of one patient as computed by the Rosetta error model (*P*<0.01) (Hughes *et al*, 2000; van 't Veer *et al*, 2002). Of these significantly expressed genes per pair, on average more than 97% were coexpressed, and 3–149 genes were antiexpressed (Supplementary Table S2), that is, upregulated in the primary tumour and downregulated in the lymph node metastasis or reciprocally, compared to a reference pool of 100 primary breast tumours. The scrutiny of the molecular functions of the differentially expressed genes in the 15 matching pairs revealed several repeating biological themes (Supplementary Table S3). On average, 18% (range 4.7–66.6%) of the contrarily expressed genes within a matching pair were extracellular-matrix and cell-matrix interaction molecules (e.g., MMP3, MMP9, osteopontin, CD44, COL1A1, L-selectin, VCAM-1, integrin alpha 2, thrombospondin 4) and 4.2% (range 0–20%) growth factors, growth factor receptors and growth factor-binding proteins (e.g., insulin-like growth factor IGF1, IGF2, t-PA, IGFBP3), as well as immune response, cell cycle and signal transduction molecules (see Supplementary Table S2). Since only approximately 1% of the 18 336 genes on the cDNA array represent genes involved in extracellular structure organisation and biogenesis, defined by the gene ontology tool ‘FatiGO’ (Al-Shahrour *et al*, 2004) (data not shown), we see a noticeable increase in this functional group of genes antiexpressed within the matching pairs. No distinct pattern of these differentially expressed genes can be identified, since different sets of these genes are upregulated in some lymph node metastases and downregulated in others compared to their matching primary breast tumours.

Matrix metalloproteases, one tissue inhibitor of metalloproteases (TIMP-3) and members of the IGF family are regularly contrarily expressed between primary tumours and lymph node metastases. The differential expression of MMP3 in primary and metastatic tumours of patient 1 and 7 and of MMP9 in patient 1 and 15 could be confirmed by quantitative real-time PCR (Supplementary Figure S4).

### Expression of genes determining the lymph node as metastatic destination of tumour cells

**Figure 4:**
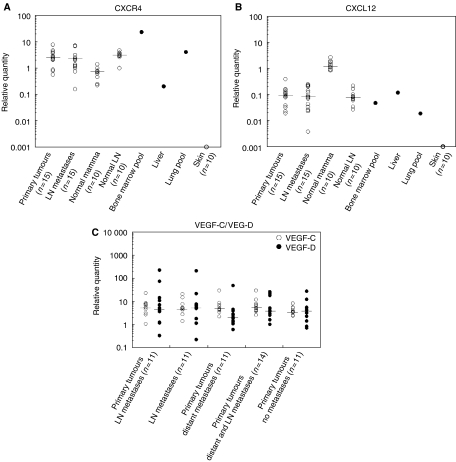
Relative quantity of expression of (**A**) CXCR4, (**B**) CXCL12 and (**C**) VEGF-C and VEGF-D in primary breast carcinomas, lymph node metastases and various normal tissues. LN=lymph node. Primary tumours LN metastases=primary breast carcinomas that developed lymph node metastases only; primary tumours distant metastases=primary breast carcinomas that developed distant metastases only; primary tumours distant and LN metastases=primary breast carcinomas that developed distant and lymph node metastases; primary tumours no metastases=primary breast carcinomas that developed no metastases. The median expression level for each marker gene within a group is indicated by a horizontal line.

When analysing the significant genes antiregulated in the individual pairs, we expected to identify chemokines, since they had been reported to be differentially expressed between primary tumours and various metastasis sites in a mouse model (Muller *et al*, 2001). However, no chemokine was differentially regulated in our matching pairs, perhaps due to changes in gene expression that are too subtle to be detected by microarrays. We subsequently determined CXCR4 and CXCL12 expressions by quantitative real-time PCR in the pairs as well as in normal tissues of the breast, lymph nodes, bone marrow, lung, liver and skin (Figure 4A and B). Still, using a more sensitive technique, we did not detect a difference in CXCR4 and CXCL12 expressions between primary breast carcinomas and matching lymph node metastases. We found the median expression of CXCR4 to be significantly higher in breast tumours, in both primary and metastatic carcinomas, than in normal mammary tissue (*P*=0.0027 and 0.016, respectively) (Figure 4A). CXCR4 was, however, even more highly expressed in normal bone marrow and normal lung, two breast cancer metastasis sites, than in the breast tumours studied (Figure 4A). CXCL12 expression was higher in breast cancer metastasis organs, normal lymph nodes, bone marrow, liver and lung, compared to skin, a site of low metastasis frequency, as described (Muller *et al*, 2001) (Figure 4B). However, CXCL12 expression was highest in normal mammary tissue, and no difference in the median CXCL12 expression between normal lymph nodes or liver and the 15 matching pairs could be observed by quantitative real-time PCR.

A second group of molecules we expected to be highly expressed in our primary breast carcinomas were the vascular endothelial growth factor genes VEGF-C and VEGF-D, as their overexpression was associated with lymph vessel neogenesis and increased lymphatic metastasis in mice (Karpanen *et al*, 2001; Mandriota *et al*, 2001; Skobe *et al*, 2001; Stacker *et al*, 2001). We determined the VEGF-C/D expression levels by quantitative real-time PCR in our matching pairs, and in primary tumours of 10 breast cancer patients who exclusively developed distant metastases, of 10 patients who developed both lymph node and distant metastases and of 11 patients who did not develop any regional or distant metastases within a median follow-up of 8.6 years. The tumours show large spread in VEGF-C/D expression (Figure 4C). No significant differences in the median expression levels of these two molecules between the different groups of primary breast carcinomas investigated were found.

### Prediction of the lymph node status

Although lymph node metastasis is a prognostic factor for disease outcome in breast cancer, it is still unknown whether metastasis to distant sites proceeds sequentially from lymph node metastasis or in parallel by a haematogenous route. The finding that expression profiles of human primary breast tumours can predict the risk of distant metastasis development, in patients with both lymph node-negative and lymph node-positive disease (van de Vijver *et al*, 2002), suggests that the molecular mechanisms underlying distant haematogenous and lymphogenic metastasis are distinct. Furthermore, in this data set, including 151 lymph node-negative and 144 lymph node-positive patients, no expression signature predicting the lymph node status could be determined (Supplementary Figure S5A and S5B). In contrast, Huang *et al* (2003) identified a gene expression pattern associated with the breast tumour's likelihood of having lymph node metastases at diagnosis. For validation, we applied this lymph node expression signature on the data set of the 295 patients described above. The classification accuracy obtained from the crossvalidation procedure for predicting the lymph node status in these patients was however only about 50% (Supplementary Figure S5C and S5D). This implies that the expression pattern illustrated (Huang *et al*, 2003) is not a general predictor of nodal metastasis in primary breast carcinomas.

## DISCUSSION

Elucidation of the molecular mechanism underlying lymph node metastasis is likely to have implications for the clinical management of breast cancer. The data presented here show that gene expression profiles of primary breast carcinomas are maintained in their lymph node metastases, which has been suggested earlier in two patients using a smaller subset of genes (Perou *et al*, 2000). In this larger study, we have not been able to identify common differentially expressed genes that discriminate the group of primary tumours from the group of lymph node metastases using two different microarray platforms. This finding is rather surprising, since we only analysed metastases from one metastasis site. Furthermore, we showed by expression profiling of two primary breast carcinomas and a metastasis obtained from the same patient that the similarity between primary and metastatic tumours can be attributed to tumour-intrinsic rather than to patient-specific factors.

We were not able to develop a classifier predicting the lymph node status in a series of 295 primary breast tumours. These data suggest that lymph node metastasis occurs independent of distant haematogenous metastasis, and therefore that the axillary lymph node status is not the most reliable predictor of disease course in breast cancer patients.

Moreover, the molecular mechanisms determining breast cancer lymph node metastasis remain poorly understood. Whether the expression of VEGF-C and VEGF-D also plays a role in lymphangiogenesis and the formation of lymph node metastases in human tumours, as described for immunodeficient mice (Karpanen *et al*, 2001; Mandriota *et al*, 2001; Skobe *et al*, 2001; Stacker *et al*, 2001), is still unknown. We did not find a correlation between the VEGF-C and/or VEGF-D expression level, determined by real-time PCR, and the formation of lymph node metastases in the human primary breast carcinomas studied. In this context, it is important to note that the results obtained with the experimental VEGF breast tumour models and the correlative clinical studies are rather inconsistent. The expression of VEGF-C in MB-435 tumours caused an increase not only of lymph node but also of lung metastases (Skobe *et al*, 2001). MCF-7-VEGF-C tumours caused lymph node metastasis in nude mice in one study (Mattila *et al*, 2002), but in another report they did not (Karpanen *et al*, 2001). In clinicopathological studies, a positive correlation between VEGF-C levels in primary breast carcinomas and lymph node metastases was observed only once (Kurebayashi *et al*, 1999; Gunningham *et al*, 2000; Kinoshita *et al*, 2001; Koyama *et al*, 2003). Vascular endothelial growth factor-D expression was shown to be associated with lymph node metastasis (Kurebayashi *et al*, 1999; Nakamura *et al*, 2003), although an inverse correlation with lymphatic invasion and the number of nodal metastases was described (Koyama *et al*, 2003). Taken together, these results indicate that the involvement of VEGF-C/D in human breast tumour lymph node metastasis is far from firmly established.

In contrast to the mammary tumours in the animal models (Karpanen *et al*, 2001; Skobe *et al*, 2001; Mattila *et al*, 2002), we did not observe intratumoral lymph vessels and only a low density in the peritumoral areas in our human invasive breast cancer (data not shown), in agreement with others (Williams *et al*, 2003). In line with this observation, no association between the presence of intratumoral lymphatic structures and the axillary nodal status or survival could be found, but between the peritumoral lymph vessel density and poor outcome in ductal breast cancer (Bono *et al*, 2004). The peritumoral lymphatics in human breast carcinomas appear to be mature pre-existing vessels rather than newly proliferating ones, as no cycling endothelial cells could be found (Williams *et al*, 2003). These findings not only reveal fundamental differences in the histology between human and mouse mammary tumours metastasising to the lymph nodes, but also suggest that human breast tumours disseminate by invasion of pre-existing peritumoral lymphatics and do not require lymph neogenesis.

The organ-specific spread of breast cancer cells to different sites, including the lymph nodes, has been reported in a mouse model to require the chemokine receptor CXCR4 on tumour cells and the chemokine CXCL12 in target organs (Muller *et al*, 2001). Using real-time PCR, we found CXCR4 expression to be significantly higher in breast carcinoma cells than in normal mammary tissue, in concordance with others (Muller *et al*, 2001; Balkwill, 2004). Our present results suggest a role for CXCR4 in breast tumorigenesis rather than in the invasion of metastasis target organs. Indeed, it has recently been shown that carcinoma-associated fibroblasts secrete CXCL12 and therewith stimulate tumour proliferation directly by acting through CXCR4 found on the breast cancer cells (Orimo *et al*, 2005).

The subtle differences in gene expression observed within the individual pairs of matching primary tumour and lymph node metastasis did not reveal one common lymph node metastasis-specific gene set. Hao *et al* (2004) also identified differences within tumour and lymph node metastasis pairs obtained from one individual, although employing a less detailed analysis. We did identify common gene groups, involved in ECM remodelling, cell-matrix interaction, growth factor signalling and immune response, to be differentially expressed between primary and metastatic tumours. Our findings might reflect the dynamic changes in tumour cell interactions with the microenvironment, and suggest that most of the subtle differences between primary breast tumours and lymph node metastases relate to the stromal component rather than to the tumour itself. An example is MMP9, which is highly expressed in the lymph node metastasis of patient 1 (1318 relative expression units), and more than 40 times lower in the metastasis of patient 15 (32 relative expression units), who in turn shows high MMP9 expression in the primary breast tumour (658 relative expression units) (Supplementary Figure S4). Based on these data, metastasising primary breast carcinomas appear to be unique and complex organs that may use individual sets of genes to accomplish lymph nodes metastasis.

In summary, based on the result that no classifier in primary breast tumours predicting the lymph node status could be identified, our data suggest a model that predicts lymph node metastasis to occur independent of distant metastasis as a random event. Further studies are needed to solve this very important issue.

## Figures and Tables

**Table 1:** Patient characteristics of 15 patients with matching primary tumours and lymph node metastases

**Patient number**	**Age at diagnosis (years)**	**Primary tumour diameter (mm)**	**Number positive LN**	**ER-*α* status**	**WHO type carcinoma**
1	77.4	30	1/14	+	IDC
3	40.5	80	12/12	−	IDC
4	70.4	45	2/8	+	Mucinous
5	66.3	18	1/18	+	IDC
6	49.0	50	2/8	−	IDC
7	65.6	18	14/14	−	IDC
8	37.6	35	6/24	−	Metaplastic
9	56.5	35	17/17	+	ILC
10	55.0	22/12^a^	16/18	+	ILC
11	49.2	35	1/17	+	IDC
12	89.0	21/24^a^	3/18	+	IDC
14	37.6	30	2/22	+	IDC
15	70.1	23	2/17	+	IDC
16	83.7	30	2/12	−	IDC
17	39.8	35/18^a^	2/14	−	IDC

a One tumour with two foci of different sizes. LN=lymph node; ER=oestrogen receptor; WHO=World Health Organization; IDC=invasive ductal carcinoma (NOS); ILC=invasive lobular carcinoma.

**Table 2:** Patient characteristics of four patients with two primary breast tumours and a metastasis of either of the two tumours

**Patient number**	**Age at diagnosis (years)**	**Primary tumour diameter (mm)**	**Number positive LN**	**ER-*α* status**	**WHO type carcinoma**
18A	55.4	12	0/10	+	IDC
18B	55.4	17	3/11	+	IDC
21A	44.9	24	2/18	−	IDC
21B	48.9	37	0/10	−	IDC
23A	66.0	36	1/13	−	IDC
23B	66.0	24	0/13	+	ILC
24A	62.9	15	0/11	−	IDC
24B	64.5	18	0/16	+	IDC

See Table 1 footnote for abbreviations.
