# Supplementary material for: No common denominator for breast cancer lymph node metastasis
Source: Br J Cancer. 2005 Sep 27;93(8):924–32. doi: 10.1038/sj.bjc.6602794 (PMC2361648; doi:10.1038/sj.bjc.6602794)
Supplement: Supplementary Information [file 93-6602794x1.pdf]

Supplementary information

A

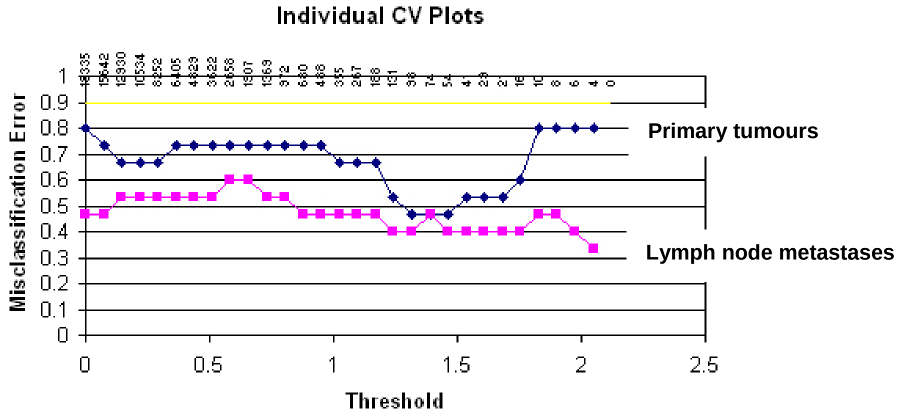

B

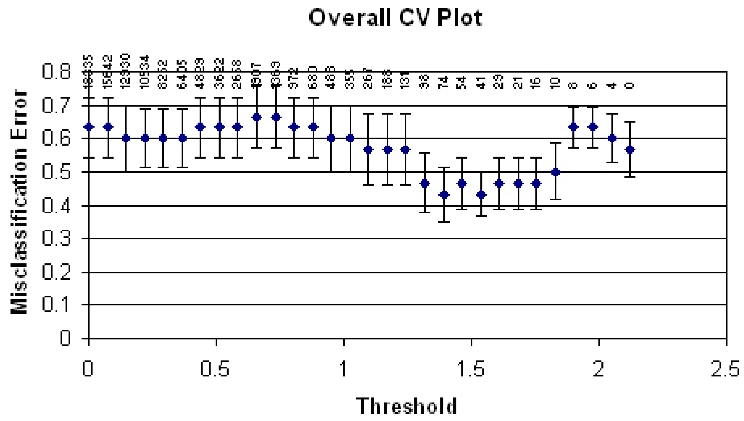

Supplementary Figure S1 Predicting analysis of microarrays (PAM). The analysis includes 15 pairs of primary tumours and lymph node metastases, using all 18,366 genes of the array. After training, a 10-fold balanced cross validation was performed. (**A)** shows the prediction accuracy for the separate classes (primary tumours (blue) and metastases (pink)). Accuracy rates are 60% for lymph node metastases (misclassification 40%) and 50% for primary tumours. (**B)** shows the overall performance (both on primary tumours and metastases) of the cross validation. The threshold depicted on the X-axis correspondence to changing the number of genes used (see top bar – horizontal). The changes of miss classification are depicted on the Y-axis. For the first analysis performance never exceeds 57% accuracy (=43% misclassification).

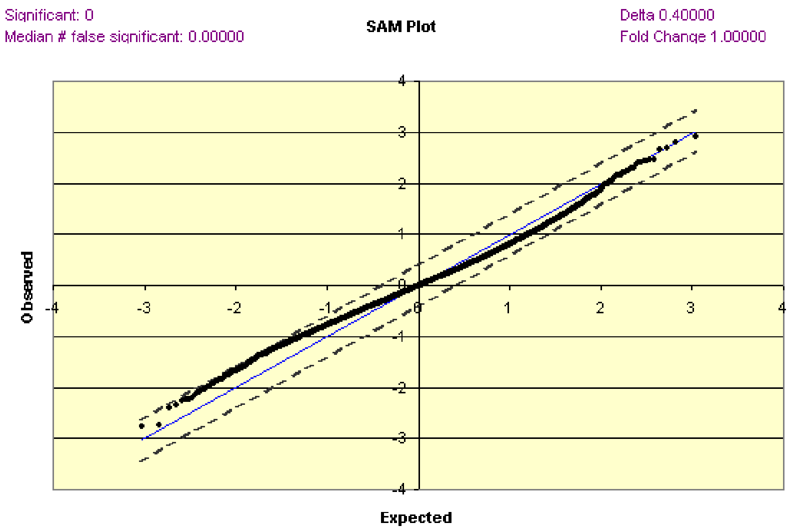

Supplementary Figure S2 Significance analysis of microarrays (SAM). The input criteria included a Delta of 0.4 and one-fold or greater expression in the primary breast tumour group as compared to the lymph node metastases group. No gene of the 18,336 genes on the cDNA array was identified as differentially expressed between the two groups.

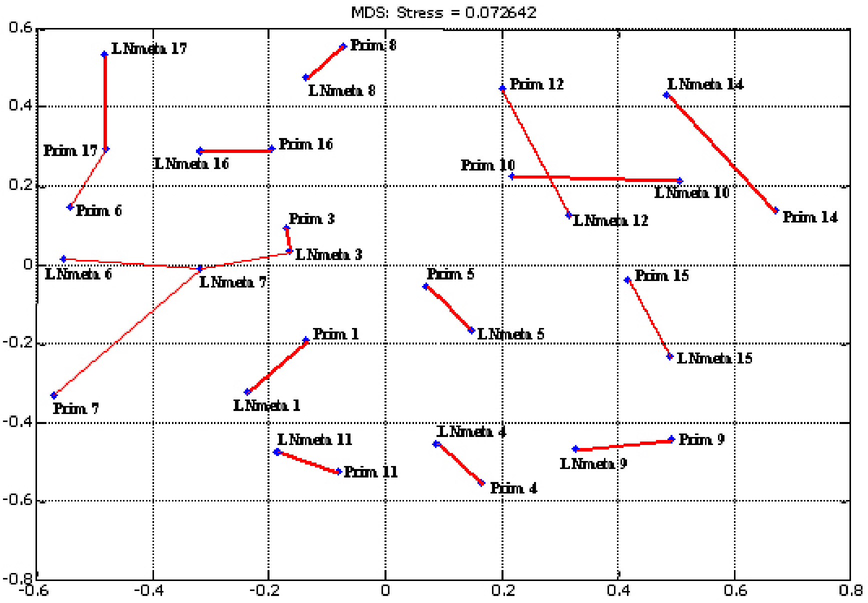

Supplementary Figure S3 Two-dimensional representation of a multidimensional scaling analysis of 15 matching primary and metastatic tumours using 18336 genes. X- and Y-axis; distance in arbitrary units. A thick red line indicates two-way-pairing, and a thin red line one-way pairing. Prim *n*, LNmeta *n* (*n* = 1-17); patient number primary tumour, patient number lymph node metastasis, respectively.

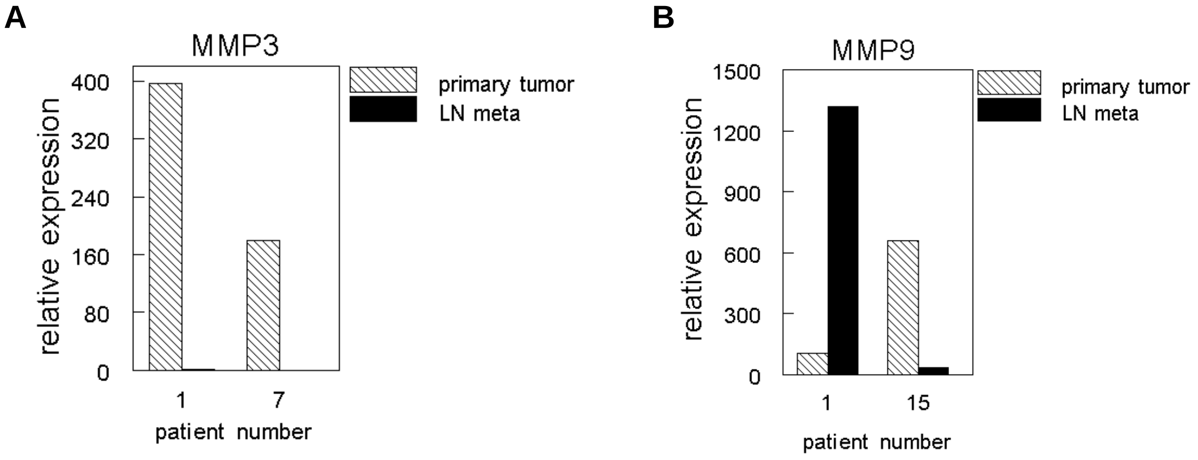

Supplementary Figure S4 Relative quantity of expression of (**A**) MMP3 and (**B**) MMP 9 of primary breast carcinomas (striped bars) and matching lymph node metastases (black bars) for patient number 1, 7 and 15. LN meta = lymph node metastasis.

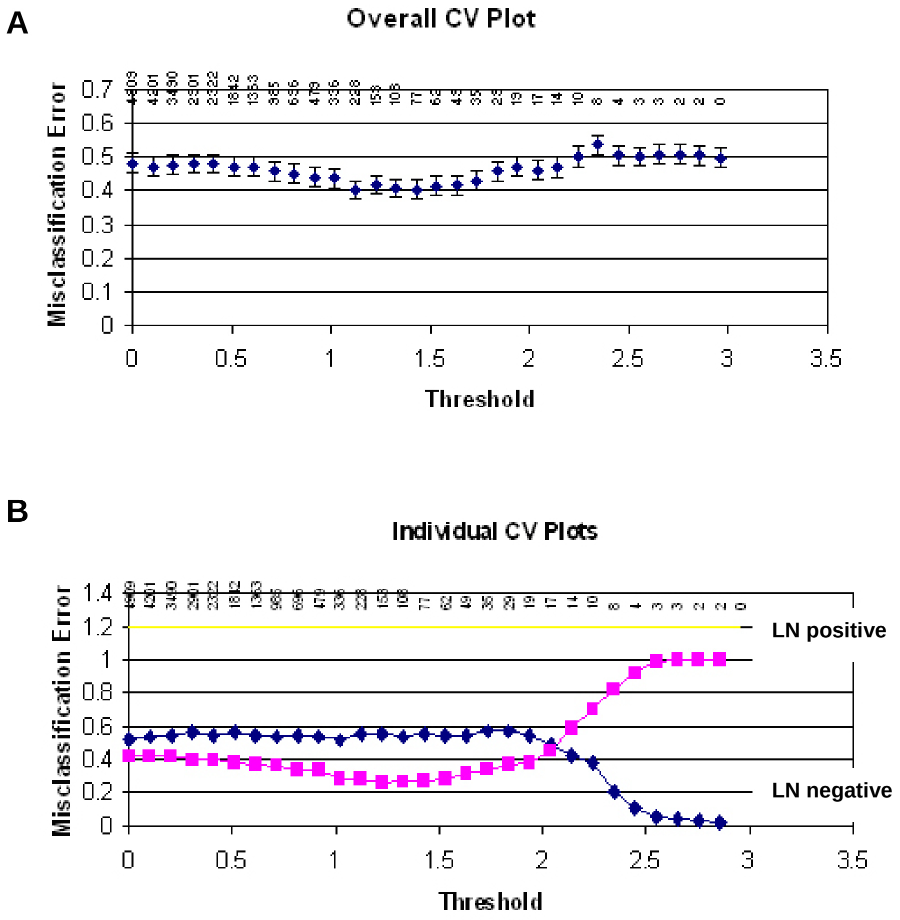

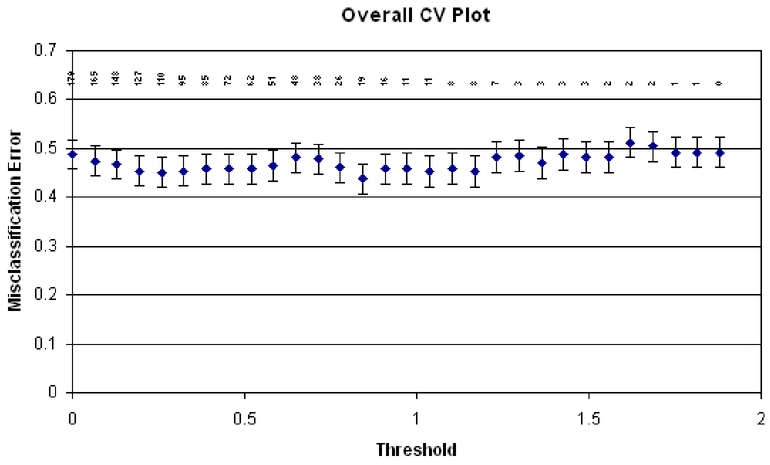

D

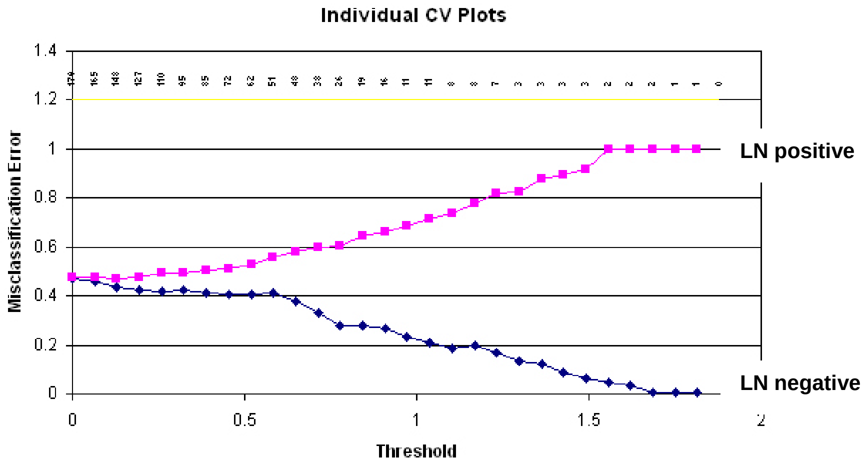

Supplementary Figure S5 Predicting analysis of microarrays (PAM). For the first analysis the most significantly expressed 5000 probes across 295 tumour samples were selected (van de Vijver *et al*, 2002) (A and B). The second analysis was done on 151 lymph node-negative and 144 lymph node-positive patients (van de Vijver *et al*, 2002) and the gene list exists of 172 probes matched from the lymph node classifier by Huang *et al*. (Huang *et al*, 2003)(C and D). (**A** and **C**) show the overall performance (predicting both LN negative and LN positive status) of the cross-validation. The threshold depicted on the X-axis corresponds to changing the number of genes used. The changes of misclassification are depicted on the Y-axis. (**A**) The overall accuracy to predict the LN status in 295 patients is 60% (0.40 misclassification). (**C**) The performance to predict the LN status in 295 patients using the classifier by Huang *et al*. never exceeds 56% accuracy (=0.44 misclassification). **B and D** show the prediction accuracy for the separate classes (LN positive (pink) and LN negative (blue)). The prediction of LN negative reaches 100% accuracy in both analyses; however at this threshold of 2.7 and 1.75, respectively, the misclassification of LN positive patients also reaches 100%. (**B**) The performance for predicting classes is stable until the reduction to 20 probes with an accuracy of 44-48% (52-56% misclassification) in the first analysis. (**D**) For the best performance in second analysis all genes are used. Accuracy rates are 52% for both classes (misclassification 48%). LN: lymph node.

Gene	Accession No.	Primers	Probe (5'FAM-3'TAMRA)
**VEGF-C**	(NM_005429)	Sense: TCAAGGACAGAAGAGACTATAAAATTTGC	ATACACACCTCCCGTGGCATGCATTG
		Antisense: ACTCCAAACTCCTTCCCCACAT	
**VEGF-D**	(AJ000185)	Sense: GTATGGACTCTCGCTCAGCAT	AAGAACTCAGTGCAGCCCTAGAGAAACG
		Antisense: AGGCTCTCTTCATTGCAACAG	
**CXCR4**	(BC020968)	Sense: GCCTTATCCTGCCTGGTATTGTC	
		Antisense: GCGAAGAAAGCCAGGATGAGGAT	
**CXCL12**	(L36034)	Sense: GGAACCTGAACCCCTGCTGTG	
		Antisense: CCATTCATTTCTGCCTTCATCA	
**MMP3**	(NM_002422)	Sense: GTTCCTGATGTTGGTCACTTCAGA	
		Antisense: TCACAATCCTGTATGTAAGGTGGGT	
**MMP9**	(J05070)	Sense: ACGCAGACATCGTCATCCAGT	AGGGATACCCGTCTCCGTGCTCCG
		Antisense: CCACAACTCGTCATCGTCGA	

Supplementary Table S1 Primer and probe sequences for real-time PCR amplification. All sequences are written 5'→3'.
