# Supplementary material for: No common denominator for breast cancer lymph node metastasis
Source: Br J Cancer. 2005 Sep 27;93(8):924–32. doi: 10.1038/sj.bjc.6602794 (PMC2361648; doi:10.1038/sj.bjc.6602794)
Supplement: Supplementary Table S2 [file 93-6602794x2.pdf]

Supplementary Table S2 Significant genes (p<0.01) anti-expressed between pairs of primary tumours and matching lymph node metastases (LNmeta).

Pair number 1

Genbank	Description	Log2ratio Primary tumour	Log2ratio LNmeta
AA424786	golgi autoantigen, golgin subfamily a, 2	0.3949	-0.6399
N39161	CD36 antigen (collagen type I receptor, thrombospondin receptor)	0.3075	-0.5119
AA487429	ATP-binding cassette, sub-family B (MDR/TAP), member 2	0.9411	-0.5231
T70586	perilipin	2.1684	-0.4353
AA455120	ESTs	-0.9577	0.6690
AA634109	Fc fragment of IgG, low affinity IIa, receptor for (CD32)	1.4537	-0.4582
W51794	matrix metalloproteinase 3 (stromelysin 1, progelatinase)	1.0410	-1.3090
AA968514	WW domain binding protein 1	0.5177	-0.5641
AA425227	matrix metalloproteinase 9 (gelatinase B, 92kD gelatinase)	-0.7585	1.0761
AI344545	serum amyloid A4, constitutive	2.3588	-1.0309
AA490837	clone HQ0477 PRO0477p	0.6417	-0.4885

Pair number 3

Genbank	Description	Log2ratio Primary tumour	Log2ratio LNmeta
AA075307	matrin 3	0.3867	-0.6202
AA489640	interferon-induced protein with tetratricopeptide repeats 1	-0.5249	1.3275
AA464526	interleukin 1 receptor, type I	1.8126	-0.6641
N91887	thymosin, beta, identified in neuroblastoma cells	0.7256	-0.7364
AA434115	chitinase 3-like 1 (cartilage glycoprotein-39)	-0.6617	0.3539
T63761	uteroglobin	-0.3829	0.6481
AA160507	keratin 5 (epidermolysis bullosa simplex)	-1.8956	0.6572
AA449715	sushi-repeat-containing protein, X chromosome	-0.7124	0.4743
R52654	cytochrome c	0.7129	-0.4110
AA129677	MKP-1 like protein tyrosine phosphatase	-0.4123	0.3849
T70586	perilipin	-1.8685	1.3001
AA157001	ESTs	-0.5017	0.3303
R26046	interleukin enhancer binding factor 3, 90kD	0.5717	-0.3487
AA099153	tissue inhibitor of metalloproteinase 3 (Sorsby fundus dystrophy)	0.4628	-0.3353
AA496149	3-hydroxy-3-methylglutaryl-Coenzyme A synthase 2 (mitochondrial)	-0.8117	0.4862
AA074446	GTP cyclohydrolase I feedback regulatory protein	-1.1477	0.5453
T95113	Homo sapiens cig5 mRNA, partial sequence	-0.5496	0.7570
AA634109	Fc fragment of IgG, low affinity IIa, receptor for (CD32)	-1.5520	1.3351
N92901	fatty acid binding protein 4, adipocyte	-1.7166	1.3456
AA486554	hypothetical protein FLJ10743	-0.5043	0.4403
AA464601	tetraspan 5	-0.6123	0.7519
AA398356	chromosome 11 open reading frame 14	-0.7592	0.4386
H04769	Homo sapiens cAMP-dependent protein kinase inhibitor beta mRNA	-1.3328	0.9677
N57557	chromosome 11 open reading frame 14	-0.8068	0.3771
H23252	hypothetical protein FLJ20533	0.7494	-0.7805
AA160751	map kinase phosphatase-like protein MK-STYX	0.9718	-0.6983
R38933	plasminogen activator, tissue	-1.0581	0.8091
AA450363	phosphatidylinositol glycan, class F	-0.3528	0.5471

AA974008	meiotic recombination (S. cerevisiae) 11 homolog A	-0.8592	0.7518
AI344545	serum amyloid A4, constitutive	-2.0227	1.7188
AI049675	GDNF family receptor alpha 1	0.5440	-0.7501
AI420743	alcohol dehydrogenase 3 (class I), gamma polypeptide	-1.4659	1.1590

Pair number 4

Genbank	Description	Log2ratio Primary tumour	Log2ratio LNmeta
AA131664	hypothetical protein FLJ20277	0.4415	-0.8294
AA775616	secreted phosphoprotein 1 (osteopontin, bone sialoprotein I)	-1.0049	0.5746
AA504356	ESTs	0.3344	-0.6856

Pair number 5

Genbank	Description	Log2ratio Primary tumour	Log2ratio LNmeta
AA485893	ribonuclease, RNase A family, 1 (pancreatic)	0.7329	-0.5170
AA598601	insulin-like growth factor binding protein 3	0.3272	-0.8430
H90899	desmoplakin (DPI, DPII)	-0.3133	0.7674
N64504	ESTs	-0.4598	0.5418
AA056013	Microfibril-associated glycoprotein-2	0.7498	-0.6443
T56281	RNA helicase-related protein	0.7267	-0.6399
AA455237	hypothetical protein FLJ20705	-0.4759	0.4551
AA404609	hypothetical protein FLJ22418	-0.8530	0.5199
AA954935	matrix metalloproteinase 11 (stromelysin 3)	0.5563	-0.6240

Pair number 6

Genbank	Description	Log2ratio Primary tumour	Log2ratio LNmeta
H69531	transferrin	-0.7171	0.8919
AA039370	Homo sapiens transcribed sequence with strong similarity to protein pir:A40032 (H.sapiens) A40032 transcription enhancer factor TEF1	-0.6426	0.7638
H74265	protein tyrosine phosphatase, receptor type, C	-0.7840	1.5902
R26186	protein phosphatase 1, catalytic subunit, beta isoform	-0.9039	0.6770
AA031514	matrix metalloproteinase 7 (matrilysin, uterine)	-0.7851	1.3873
AA143331	matrix metalloproteinase 1 (interstitial collagenase)	-0.9058	0.5769
N62847	lysosomal-associated membrane protein 2	-0.4645	0.4790
H20822	Fc fragment of IgG, low affinity IIIb, receptor for (CD16)	-0.3142	1.8526
W67174	Homo sapiens integrin, beta 1 (fibronectin receptor) (ITGB1), mRNA	-0.8275	0.4998
H11482	interferon gamma receptor 1	-0.9255	0.8573
H18070	mitochondrial translational initiation factor 2	-1.0642	0.8784
H77652	GATA binding protein 6	-0.7533	0.7429
W58032	frizzled-related protein	-1.0860	0.8707
R06567	phosphoinositide-3-kinase, regulatory subunit, polypeptide 3 (p55)	-0.4781	0.9349
W02101	heterogeneous nuclear ribonucleoprotein A2/B1	-0.7193	0.9115
H65660	acyl-Coenzyme A oxidase 1, palmitoyl	-0.2806	0.5274
AA029308	mature T-cell proliferation 1	-0.5997	0.6355
AA490466	gap junction protein, beta 2, 26kD (connexin 26)	-0.4964	0.6364
R93124	aldo-keto reductase family 1, member C1	-0.4313	1.1175
H23187	carbonic anhydrase II	0.5471	-0.3627
R05278	UDP-N-acetyl-alpha-D-galactosamine	-0.6021	0.3940

AA478589	apolipoprotein E	-1.4286	0.5499
H63077	annexin A1	-1.7623	0.6122
H16591	vascular cell adhesion molecule 1	-0.5842	0.6715
H65676	suppression of tumorigenicity 13 (Hsp70-interacting protein)	-0.6314	0.7034
AA279804	RAP1A, member of RAS oncogene family	-0.4130	0.9240
AA088745	RAB6A, member RAS oncogene family	-0.5452	1.0175
AA457042	myxovirus (influenza) resistance 1, homolog of murine	0.7186	-1.1522
AA488073	mucin 1, transmembrane	1.0070	-0.7388
AA465366	leukotriene A4 hydrolase	-0.6039	0.7574
AA150828	mitogen-activated protein kinase kinase kinase 5	-0.5134	0.3486
AA459866	KIAA0332 protein	-0.7906	0.6665
AA490996	interferon, gamma-inducible protein 16	-0.7780	1.2922
AA598601	insulin-like growth factor binding protein 3	-0.6276	0.7386
AA441930	phosphatidylinositol binding clathrin assembly protein	-0.9221	0.7705
AA449753	capping protein (actin filament) muscle Z-line, alpha 1	-0.9085	0.7668
AA449975	novel RGD-containing protein	-0.6270	0.4378
AA279762	N-myc (and STAT) interactor	-0.5026	0.3908
AA446028	paraoxonase 2	-0.5374	0.6086
AA447551	RBP1-like protein	-0.5419	0.6935
AA463498	immunoglobulin (CD79A) binding protein 1	-0.5791	0.8441
AA487914	hydroxysteroid (17-beta) dehydrogenase 4	-0.6942	0.2763
AA425687	DEAD/H (Asp-Glu-Ala-Asp/His) box polypeptide 1	-0.9904	0.6903
AA504351	zinc finger protein 146	-0.9191	0.7537
AA448157	cytochrome P450, subfamily I (dioxin-inducible), polypeptide 1	-1.2112	1.3876
AA463492	cytochrome b-245, beta polypeptide (chronic granulomatous disease)	-0.7567	1.2846
AA283090	CD44 antigen (homing function and Indian blood group system)	-0.4543	1.4448
N71003	programmed cell death 4	-1.5455	0.4289
N57773	Homo sapiens mRNA for KIAA1771 protein, partial cds	-0.5117	0.4534
R65792	uncharacterized hypothalamus protein HCDASE	-1.0558	1.5290
N54338	B7 homolog 3	0.8176	-0.4326
T96603	hypothetical protein FLJ14153	-0.4667	1.5745
W86653	FK506-binding protein 5	-0.9928	0.9973
N54265	oxysterol binding protein-like 1	0.3504	-0.3947
N95381	APG5 (autophagy 5, S. cerevisiae)-like	-0.6396	0.5837
R63694	ESTs	-0.4365	0.8872
H73961	actin related protein 2/3 complex, subunit 3 (21 kD)	-0.4509	0.6801
R33103	KIAA0610 protein	-0.6468	0.7344
N89738	Arg/Abl-interacting protein ArgBP2	0.3405	-0.7858
AA454617	Homo sapiens mRNA; cDNA DKFZp434E2023	-0.4171	0.4455
N36402	hypothetical protein PRO2032	-0.7709	0.6600
N20593	hypothetical protein FLJ13194	-0.5604	0.5228
AA032221	six transmembrane epithelial antigen of the prostate	-0.7394	0.4274
H94739	DKFZP566C0424 protein	-0.5334	0.3669
AA043501	v-maf musculoaponeurotic fibrosarcoma (avian) oncogene homolog	-0.9306	0.5765
AA488084	Homo sapiens, clone MGC	-0.5722	0.5486
AA682631	protein phosphatase 3 (formerly 2B), catalytic subunit, alpha isoform (calcineurin A alpha)	-0.5309	0.2919
AA677388	inter-alpha (globulin) inhibitor, H1 polypeptide	-2.8836	2.7280
AA496628	non-metastatic cells 2, protein (NM23B) expressed in	-1.1537	0.4500
AA677706	lactotransferrin	-3.2133	3.2339

AA701545	ribonuclease, RNase A family, k6	-0.3171	1.2603
AA670438	ubiquitin carboxyl-terminal esterase L1 (ubiquitin thiolesterase)	0.6815	-0.5424
AA485371	bone marrow stromal cell antigen 2	-0.7697	0.7215
H88599	predicted osteoblast protein	-0.3954	0.3784
H87471	kynureninase (L-kynurenine hydrolase)	-1.0687	1.1034
AA430675	Fanconi anemia, complementation group G	0.5724	-0.4100
AA683085	high-mobility group (nonhistone chromosomal) protein 1	-0.4660	0.3804
AA634028	Human mRNA for SB classII histocompatibility antigen alpha-chain	-0.7656	1.6982
N29844	peptidase (mitochondrial processing) beta	-0.7969	0.7632
AA496097	heterogeneous nuclear protein similar to rat helix destabilizing protein	-0.3582	0.6531
AA680136	coagulation factor V (proaccelerin, labile factor)	-0.7845	1.4428
AA625995	zinc finger protein 9 (a cellular retroviral nucleic acid binding protein)	-0.5392	0.7445
AA446907	Homo sapiens CDA02 mRNA, complete cds	-0.9636	1.1065
AA669674	eukaryotic translation initiation factor 3, subunit 6 (48kD)	-0.6458	0.5473
W93520	hypothetical protein FLJ13194	-0.6201	0.6264
AA186804	ERO1 (S. cerevisiae)-like	-0.5545	0.3614
AA186327	NS1-associated protein 1	-0.7583	0.6548
AA069704	6.2 kd protein	-0.4776	1.1644
N22924	disabled (Drosophila) homolog 1	-0.5715	0.5673
AA400292	disabled (Drosophila) homolog 2 (mitogen-responsive phosphoprotein)	-0.8506	0.6069
AA032205	hypothetical protein FLJ10853	-0.7807	0.6819
AA454668	prostaglandin-endoperoxide synthase 1	-0.4498	0.9303
AA454969	hypothetical protein DKFZp586F1122 similar to axotrophin	-1.0399	0.5906
AA424568	ADP-ribosylation factor-like 5	-1.0036	0.5752
AA495835	erythrocyte membrane protein band 4.1-like 3	-1.1704	0.5626
AA456302	hypothetical protein DKFZp547A023	-0.3270	0.8305
AA450334	hypothetical protein FLJ20481	-0.3195	0.5473
AA451886	EST	-1.4951	1.4516
N74285	CDC5 (cell division cycle 5, S. pombe, homolog)-like	-0.7661	0.4204
W76339	nuclear factor (erythroid-derived 2)-like 3	-0.5488	0.3906
AA775616	secreted phosphoprotein 1 (osteopontin, bone sialoprotein I)	-1.3832	1.0808
H98619	LCHN protein	-0.7575	0.9964
N34436	v-maf musculoaponeurotic fibrosarcoma (avian) oncogene homolog	-0.6752	0.6186
N34316	protein phosphatase 1, regulatory (inhibitor) subunit 1B (DARPP-32)	1.0592	-1.7652
N45236	ESTs	1.3832	-0.3424
N91588	Homo sapiens cDNA	-0.7467	0.5611
AA417761	Homo sapiens clone 24416 mRNA sequence	-0.8181	1.1202
AA629027	hypothetical protein FLJ23293 similar to ARL-6 interacting protein-2	-0.3782	0.6335
AA489210	CGI-07 protein	-1.5057	0.3670
AA504164	hypothetical protein FLJ11273	-0.7640	0.4908
AA677432	phospholipase C, epsilon 2	-0.5907	0.7554
AA864554	S100 calcium-binding protein A9 (calgranulin B)	-1.1119	3.6683
AA970720	KIAA0592 protein	-0.5895	0.5019
AI002301	RAB13, member RAS oncogene family	-0.5277	0.6388
AA705221	hypothetical protein FLJ10587	0.4818	-1.1818
AA488899	KIAA0916 protein	-0.5231	0.5345
H23211	hypothetical protein MGC3077	-1.2163	0.5975
AA971543	apolipoprotein L, 3	-0.5214	0.9138
AA969785	Homo sapiens cDNA FLJ20667 fis, clone KAIA596	-0.4971	0.5466

N74203	delta-like homolog (Drosophila)	1.3500	-0.6044
AA454111	frizzled-related protein	-0.8903	0.8882
AA630549	major histocompatibility complex, class II, DR beta 1	-0.8581	0.7280
AI141490	pentaxin-related gene, rapidly induced by IL-1 beta	-0.6476	0.6304
AI218900	H4 histone family, member I	-0.3011	0.5464
AI344545	serum amyloid A4, constitutive	-1.0704	2.9974
AI360840	SWAP-70 protein	-0.9124	0.4467
AI360356	synaptophysin	0.7664	-0.7543
AI039001	DKFZP586D211 protein	-0.7175	0.4149
AI206454	fatty-acid-Coenzyme A ligase, long-chain 3	-0.6981	0.4474
AI669320	differentially expressed in hematopoietic lineages	-1.9614	5.1000

Pair number 7

Genbank	Description	Log2ratio Primary tumour	Log2ratio LNmeta
AA478268	C-terminal binding protein 1	-0.4337	0.2973
N68467	peroxiredoxin 5	-0.4042	0.4388
AA434390	hypothetical protein PRO0899	-0.4368	0.3796
N31808	ARP1 (actin-related protein 1, yeast) homolog A (centractin alpha)	0.9721	-1.4020
AA457700	stearoyl-CoA desaturase (delta-9-desaturase)	0.4438	-0.3511
T56281	RNA helicase-related protein	0.3383	-0.7783
W93370	killer cell lectin-like receptor subfamily C, member 3	0.2814	-0.5090
R44647	ESTs, Weakly similar to ALU1_HUMAN ALU SUBFAMILY J	0.3080	-0.4390
W51794	matrix metalloproteinase 3 (stromelysin 1, progelatinase)	0.3272	-1.2857
AA042812	hypothetical protein FLJ10326	-0.4550	0.4249
T63245	ESTs	-0.5354	0.3268
W38387	Homo sapiens mRNA; cDNA DKFZp564O2364	0.4319	-0.6962
AI000285	RAB11B, member RAS oncogene family	-0.4097	0.3952
AA989502 |	ESTs	0.4207	-0.3984
AA496376	solute carrier family 25 (adenine nucleotide translocator), member 6	-0.5092	0.4146
AI344545	serum amyloid A4, constitutive	0.3366	-0.5505
AI361330	methylene tetrahydrofolate dehydrogenase (NAD+ dependent),	-0.4041	0.4586
AI147534	glutathione peroxidase 3 (plasma)	-0.3655	0.4476
AI241434	ESTs, Highly similar to inositol 1,3,4-trisphosphate 5/6-kinase	-0.3788	1.0246

Pair number 8

Genbank	Description	Log2ratio Primary tumour	Log2ratio LNmeta
H00662	selectin L (lymphocyte adhesion molecule 1)	-0.6345	0.6459
R32848	S100 calcium-binding protein P	0.6732	-0.4671
T61271	phospholipase A2, group IIA (platelets, synovial fluid)	-0.5820	1.0751
AA489498	WW Domain-Containing Gene	-0.5207	0.6263
AA598817	preferentially expressed antigen in melanoma	-1.1780	0.6403
AA598478	complement component 7	-1.5463	0.5428
T86934	CD79A antigen (immunoglobulin-associated alpha)	-0.6421	0.6980
T67053	immunoglobulin lambda locus	-2.8167	1.8789
H04382	wingless-type MMTV integration site family member 2	-0.6490	0.4623
W73140	kallikrein 5	-1.6551	0.4722
R16098	lipopolysaccharide-binding protein	-0.4589	1.2060

AA459935	Homo sapiens cDNA	0.3788	-0.4088
N78828	wingless-type MMTV integration site family member 2	-0.6493	0.4123
T64192	Homo sapiens TCR BV3 mRNA for T cell receptor beta chain (CDR3)	-0.4474	0.6615
AA406020	interferon-stimulated protein, 15 kDa	-1.0626	1.0328
W73790	immunoglobulin lambda-like polypeptide 1	-2.7401	0.8058
AA485371	bone marrow stromal cell antigen 2	-0.6498	0.6011
H87471	kynureninase (L-kynurenine hydrolase)	-0.4423	1.2416
AA669674	eukaryotic translation initiation factor 3, subunit 6 (48kD)	0.3946	-0.5785
AA194043	bradykinin receptor B2	-1.5796	0.6363
AA455896	glypican 1	-1.1130	0.7925
AA863383	pim-2 oncogene	-0.8283	0.8347
AA876021	cytochrome b-245, alpha polypeptide	-0.4543	0.6803
R34568	2'-5'oligoadenylate synthetase 2	-0.5576	0.9703
AA939088	hypothetical protein FLJ10781	0.7812	-0.5823
AA146773	2',5'-oligoadenylate synthetase 1 (40-46 kD)	-0.5567	0.9428
AA206042	variable charge, X chromosome	-0.5789	1.6104
AI299994	immunoglobulin kappa variable 3D-15	-2.4014	0.8303
AA913206	G antigen 5	-0.3354	1.9410
AA971714	Clone BCSynL38 immunoglobulin lambda light chain variable region	-1.2568	0.8005
AA490837	clone HQ0477 PRO0477p	-1.8672	0.9645
AI654630	immunoglobulin heavy constant mu	-1.4879	0.5818
AI669320	differentially expressed in hematopoietic lineages	-0.6304	0.6366
AI688230	peptidyl arginine deiminase, type II	-1.0524	0.4837
AI357590	2'-5'oligoadenylate synthetase 3	-0.4533	0.4201

Pair number 9

Genbank	Description	Log2ratio Primary tumour	Log2ratio LNmeta
H63077	annexin A1	0.5062	-0.7493
AA485893	ribonuclease, RNase A family, 1 (pancreatic)	0.5145	-0.4818
AA496795	intersectin 1 (SH3 domain protein)	0.4993	-0.3340
AA504891	crystallin, alpha B	0.3461	-0.7039
W73366	ESTs	0.5824	-0.3668
N74623	insulin-like growth factor 2 (somatomedin A)	0.7014	-0.3755
H87536	collagen, type XVII, alpha 1	0.4416	-0.7308
R97095	T-cell leukemia/lymphoma 1A	-0.4008	0.8051
AA399633	ESTs	0.7058	-0.5894
AA456306	ESTs	0.3454	-0.3582
AA777187	cysteine-rich, angiogenic inducer, 61 (IGFBP10)	0.9953	-0.5372
H21977 |	ESTs, Weakly similar to CP4Y_HUMAN CYTOCHROME P450 4A11	1.3204	-0.5461
R59136	Ig superfamily protein	0.3593	-0.3806
T74255	hypothetical protein MGC10993	0.5485	-0.5935
H91046	H.sapiens gene from PAC 426I6, similar to syntaxin 7	0.8649	-1.1165
AA931725	secreted modular calcium-binding protein 2	0.3744	-0.3641
AA974008	meiotic recombination (S. cerevisiae) 11 homolog A	0.5151	-0.9498
AI351740	lymphotoxin beta (TNF superfamily, member 3)	-0.4562	2.0023
N69062	insulin-like growth factor 1 (somatomedin C)	0.6176	-0.4429
AA012944	cysteine-rich, angiogenic inducer, 61 (IGFBP10)	0.8379	-0.4064

Pair number 10

Genbank	Description	Log2ratio Primary tumour	Log2ratio LNmeta
H64347	syndecan 2 (heparan sulfate proteoglycan 1, cell surface-associated)	-0.3632	0.3482
W96155	v-jun avian sarcoma virus 17 oncogene homolog	0.3173	-0.3761
H51766	KIAA1051 protein	1.1638	-1.0353
T70337	ESTs, Highly similar to Multidrug resistance protein MRP1	1.2527	-1.0277
AA464691	DKFZP564I1922 protein	-0.4863	0.6034
AA633882	CD63 antigen (melanoma 1 antigen)	0.3801	-0.3698
AA464246	major histocompatibility complex, class I, C	0.3609	-0.4489
AA680186	small inducible cytokine subfamily A (Cys-Cys), member 19	0.5292	-0.4028
AA428959	cyclin G associated kinase	0.6163	-0.4175
H86554	ceruloplasmin (ferroxidase)	1.6517	-0.7502
AA432066	sarcoglycan, epsilon	0.8362	-0.5434
R40057	prominin (mouse)-like 1	0.6012	-0.7094
N51577	KIAA0608 protein	-0.4588	0.3599
AI004331	major histocompatibility complex, class II, DQ beta 1	0.6262	-0.3296
AA918982	Homo sapiens cDNA	1.2932	-0.7938
AI299994	immunoglobulin kappa variable 3D-15	1.2810	-0.3306
AI351740	lymphotoxin beta (TNF superfamily, member 3)	0.4825	-0.8348
AA490837	clone HQ0477 PRO0477p	0.9468	-0.8425
AI417982	cartilage intermediate layer protein, nucleotide pyrophosphohydrolase	-0.5023	1.5551

Pair number 11

Genbank	Description	Log2ratio Primary tumour	Log2ratio LNmeta
H42728	major histocompatibility complex, class II, DM alpha	-0.8768	0.3933
AA457026	KIAA0071 protein	0.4401	-0.5456
H57494	protein kinase H11; small stress protein-like protein HSP22	-0.7870	0.6277
T77840	discs, large (Drosophila) homolog 5	0.5797	-0.6092
W04668	ESTs, Weakly similar to SKD1_HUMAN SKD1 PROTEIN	0.4698	-0.5227
R76554	matrix Gla protein	0.8592	-0.3880
AA459935	Homo sapiens cDNA	0.3913	-0.6213
AA460282	signal transduction protein (SH3 containing)	-0.6545	0.8325
N47113	PRO2000 protein	0.4580	-0.6403
N74332	ESTs	-0.4781	0.3211
R91570	signal transducer and activator of transcription 4	-0.6997	0.4889
AA630082	cyclin-dependent kinase inhibitor 1B (p27, Kip1)	1.0860	-0.7596
AA677403	glycoprotein hormones, alpha polypeptide	-0.6947	0.9854
H29215	fatty-acid-Coenzyme A ligase, long-chain 3	0.6527	-0.5289
AA418903	cell cycle progression 2 protein	0.4089	-0.4523
AA495803	ESTs	0.6140	-0.4727
AA460225	hypothetical protein FLJ14007	0.4179	-0.5272
AA489232	ATPase, H+ transporting, lysosomal membrane sector associated protein	0.4470	-0.5956
AA504652	UDP-Gal	0.4682	-0.3731
AA598796	transforming, acidic coiled-coil containing protein 1	0.5878	-0.6972
AA460374	matrix Gla protein	0.4972	-0.3935
AA478949	discs, large (Drosophila) homolog 5	0.6476	-0.5410
W16836	major histocompatibility complex, class II, DQ alpha 1	-1.8510	0.3691

Pair number 12

Genbank	Description	Log2ratio Primary tumour	Log2ratio LNmeta
AA458982	sodium channel, nonvoltage-gated 1 alpha	0.5252	-0.3843
AA054073	carcinoembryonic antigen-related cell adhesion molecule 6	2.1148	-0.5556
AA463257	integrin, alpha 2 (CD49B, alpha 2 subunit of VLA-2 receptor)	1.2787	-0.6531
T95052	caspase 1, apoptosis-related cysteine protease (interleukin 1)	1.4545	-0.7886
W72748	guanylate binding protein 2, interferon-inducible	0.3439	-0.7504
N62620	potassium channel, subfamily K, member 1 (TWIK-1)	0.8833	-0.5968
H72937	2,4-dienoyl CoA reductase 1, mitochondrial	0.4281	-0.6021
H08820	isopentenyl-diphosphate delta isomerase	0.5107	-0.3809
AA456298	H2B histone family, member Q	1.9007	-0.4600
AA130633	SRB7 (suppressor of RNA polymerase B, yeast) homolog	0.5493	-0.8392
AA458472	major histocompatibility complex, class II, DQ beta 1	0.4553	-0.8901
N63845	phytanoyl-CoA hydroxylase (Refsum disease)	0.6578	-0.3461
T74567	complement factor H related 3	1.1557	-0.5511
H42728	major histocompatibility complex, class II, DM alpha	1.0576	-0.4829
AA446928	v-erb-b2 avian erythroblastic leukemia viral oncogene homolog 2	-0.7993	1.5992
AA504348	topoisomerase (DNA) II alpha (170kD)	-0.3086	0.9158
AA487893	transmembrane 4 superfamily member 1	0.4333	-1.1775
AA151486	phosphoribosyl pyrophosphate synthetase 2	0.4613	-0.4805
AA598561	CD164 antigen, sialomucin	0.4424	-0.5452
AA457042	myxovirus (influenza) resistance 1, homolog of murine	1.2613	-0.6971
AA482231	myristoylated alanine-rich protein kinase C substrate (MARCKS)	1.1043	-0.6276
AA458472	major histocompatibility complex, class II, DQ beta 1	0.4886	-0.7734
T63324	major histocompatibility complex, class II, DQ alpha 1	1.5610	-0.6046
AA497051	sialyltransferase	-0.6286	1.0426
AA598817	preferentially expressed antigen in melanoma	1.9654	-0.9511
AA485959	keratin 7	0.8925	-1.2354
AA460727	adaptor-related protein complex 3, sigma 1 subunit	0.4829	-0.7718
AA452933	H2A histone family, member L	1.0211	-0.3835
AA431770	Human Chromosome 16 BAC clone CIT987SK-A-362G6	-0.6816	0.6247
AA521490	KIAA0430 gene product	-0.5320	0.5051
W32272	IQ motif containing GTPase activating protein 2	0.5286	-0.3222
AA258396	pleckstrin homology-like domain, family A, member 1	0.9695	-0.8033
AA488609	nucleoporin 88kD	1.0457	-0.8670
AA448157	cytochrome P450, subfamily I (dioxin-inducible), polypeptide 1	1.6242	-0.9011
AA599177	cystatin C (amyloid angiopathy and cerebral hemorrhage)	0.3795	-0.3292
AA598478	complement component 7	0.6421	-1.0617
T62048	complement component 1, s subcomponent	0.4278	-0.5123
N66737	collagen, type II, alpha 1 (primary osteoarthritis, congenital)	-0.5891	0.9896
AA456931	cytochrome c oxidase subunit VIc	0.7048	-1.0457
AA283090	CD44 antigen (homing function and Indian blood group system)	0.3761	-0.6904
T63324	major histocompatibility complex, class II, DQ alpha 1	1.7676	-0.5771
T67053	immunoglobulin lambda locus	0.8151	-1.5009
R60847	TRK-fused gene	0.7067	-0.3033
AA031284	src homology three (SH3) and cysteine rich domain	0.8143	-0.3534
R63543	p75NTR-associated cell death executor; ovarian granulosa cell protein	0.9589	-1.6605
R65573	hypothetical protein	-0.2991	0.4237

N74362	serologically defined colon cancer antigen 3	-0.3532	0.4493
R31679	Homo sapiens cDNA FLJ20767 fis, clone COL06986	0.6010	-0.5319
H20138	RAB6A, member RAS oncogene family	-0.8741	1.8432
AA454862	CGI-135 protein	-0.4506	0.4556
AA485449	Homo sapiens RAB39 (RAB39) mRNA, complete cds	-0.8779	0.7860
AA609598	tyrosine 3-monooxygenase/tryptophan 5-monooxygenase activation	1.5206	-0.5759
AA459935	Homo sapiens cDNA	-0.4210	0.6803
AA453994	NPD007 protein	-0.3308	0.5797
AA464578	Homo sapiens, clone MGC	0.9359	-0.3912
AA464180	uncharacterized hypothalamus protein HBEX2	0.3925	-1.0012
N27145	L1 cell adhesion molecule (hydrocephalus, spastic paraplegia 1)	1.8198	-0.4007
W72167	ESTs	-0.4576	1.0861
N21237	Homo sapiens cDNA FLJ13545 fis, clone PLACE1006867	-0.6575	0.7289
AA010223	H2B histone family, member Q	0.9798	-0.4560
AA683073	synaptotagmin I	0.3728	-0.3538
N53959	Rhesus blood group, CcEe antigens	-1.1056	1.0677
AA633882	CD63 antigen (melanoma 1 antigen)	0.5138	-0.8486
R63647	prolactin receptor	0.7041	-0.3550
AA670439	Norrie disease (pseudoglioma)	2.6096	-0.6139
AA682423	monoamine oxidase B	-2.0035	1.4937
AA011096	monoamine oxidase A	-0.9018	0.5977
AA155913	matrix Gla protein	-1.8219	1.6164
AA485739	major histocompatibility complex, class II, DR beta 3	0.8056	-0.8517
AA702254	major histocompatibility complex, class II, DN alpha	0.6461	-0.5433
AA664195	major histocompatibility complex, class II, DR beta 1	0.3641	-0.8429
H53703	growth factor receptor-bound protein 7	-0.6807	0.8168
AA485371	bone marrow stromal cell antigen 2	0.5437	-0.5539
H87471	kynureninase (L-kynurenine hydrolase)	0.7600	-1.0295
AA485442	hypothetical protein	0.7834	-0.6024
AA152347	glutathione S-transferase A4	0.7090	-0.5190
AA626370	heme oxygenase (decycling) 2	0.5818	-0.7997
AA634028	Human mRNA for SB classII histocompatibility antigen alpha-chain	0.4889	-1.1223
AA134871	fibulin 1	1.0048	-0.6620
H86554	ceruloplasmin (ferroxidase)	1.7536	-1.7494
AA677185	ankyrin 3, node of Ranvier (ankyrin G)	1.0269	-0.3960
AA430052	KIAA1488 protein	0.4900	-0.6113
H08674	ESTs, Moderately similar to ALU4_HUMAN ALU SUBFAMILY SB2	-0.4963	0.5459
AA191461	phosphoinositide-3-kinase, catalytic, beta polypeptide	0.5902	-0.7268
AA644092	non-metastatic cells 1, protein (NM23A) expressed in	0.6395	-0.4265
AA159620	ecotropic viral integration site 2B	0.5216	-0.3564
H96654	pp21 homolog	1.2055	-0.6071
AA056377	ESTs	-1.0199	0.4470
H87106	transmembrane 4 superfamily member 6	0.5383	-0.4151
AA486288	KIAA0663 gene product	-0.4478	0.5325
AA608707	kinesin family member 5B	-0.3345	0.8511
AA120866	ESTs	-0.7243	0.4086
AA487054	ESTs, Weakly similar to ALU4_HUMAN ALU SUBFAMILY SB2	-0.5465	0.5719
AA425437	immunoglobulin superfamily, member 3	-0.5194	0.7458
AA460254	EST	-0.8538	0.5644

AA398356	chromosome 11 open reading frame 14	-1.1213	0.5135
AA436565	Homo sapiens cDNA	0.9270	-0.7513
AA479967	ESTs	0.4397	-0.4982
AA451886	EST	1.4733	-0.7745
AA453170	hypothetical protein FLJ14299	-0.7752	0.6445
AA194043	bradykinin receptor B2	0.6839	-1.0995
H70775	ESTs	0.6771	-0.4767
N71982	H2B histone family, member A	1.0907	-0.9810
AA044307	fatty acid binding protein 3, muscle and heart (mammary-derived growth inhibitor)	0.8691	-0.7162
R34568	2'-5'oligoadenylate synthetase 2	0.7278	-0.4755
R37817	hypothetical protein FLJ11269	0.7179	-0.6817
H03978	sphingosine-1-phosphatase	-0.9905	0.6278
H99120	ESTs	-0.5156	0.4512
N66158	cytochrome c oxidase subunit VIc	0.7350	-0.9855
N57557	chromosome 11 open reading frame 14	-0.9359	0.7428
N50654	Homo sapiens cDNA	0.9307	-0.7800
AA489232	ATPase, H+ transporting, lysosomal membrane sector associated protein	0.4935	-0.4047
AA521384	up-regulated by BCG-CWS	0.5468	-0.5662
AA148505	L13 protein	0.4153	-0.7181
AA629027	hypothetical protein FLJ23293 similar to ARL-6 interacting protein-2	0.6183	-0.5140
AA676625	DKFZP434G032 protein	0.6488	-1.9715
H50623	major histocompatibility complex, class II, DR beta 1	1.2658	-0.6803
AA995282	four and a half LIM domains 2	0.7010	-0.5827
AA115054	Homo sapiens clone 24775 mRNA sequence	0.4829	-0.3804
AA670383	KIAA0551 protein	0.4275	-0.5367
R38885	ESTs	0.4597	-0.5777
R38703	ESTs	-0.7465	0.8580
R38260	RAB2, member RAS oncogene family	0.3128	-0.3158
H08424	HSKM-B protein	0.4062	-0.9388
AA705221	hypothetical protein FLJ10587	1.9401	-0.5786
AA705142	H2B histone family, member Q	2.0271	-0.5101
AA626362	EST	0.7421	-0.8209
R69522 |	Homo sapiens clone 25194 mRNA sequence	-0.3204	0.4199
AA669152	KIAA0310 gene product	-0.3753	0.4822
AA918982	Homo sapiens cDNA	1.8460	-0.7817
AA939088	hypothetical protein FLJ10781	0.8141	-0.7659
AA933892	ESTs	-0.5700	0.6699
AA630549	major histocompatibility complex, class II, DR beta 1	1.4978	-0.4380
W85807	paired basic amino acid cleaving system 4	-0.6295	1.0154
AA776176	gamma-aminobutyric acid (GABA) A receptor, alpha 1	-0.3082	0.3838
AA954935	matrix metalloproteinase 11 (stromelysin 3)	-0.5658	0.3955
AA704242	serine (or cysteine) proteinase inhibitor, clade A, member 3	-0.4103	0.5754
AI076718	H2B histone family, member R	0.6660	-0.5929
W16836	major histocompatibility complex, class II, DQ alpha 1	0.7487	-1.3362
AA181643	reticulocalbin 1, EF-hand calcium binding domain	1.3945	-0.3347
AA147186	putative NFkB activating protein 373	0.6227	-0.4287
AA455942	Homo sapiens mRNA; cDNA DKFZp434D2111	0.5913	-0.6094
AA682402	major histocompatibility complex, class II, DR beta 1	1.1518	-0.6625
AA158584	calpastatin	1.2940	-0.5027

AA158236	hypothetical protein FLJ13052	1.3606	-0.6356
AI214586	major histocompatibility complex, class II, DQ alpha 1	1.1205	-0.7372
AI654630	immunoglobulin heavy constant mu	0.9828	-1.2210
AI675311	tryptase beta 1	0.3567	-0.9292
AI688440	diazepam binding inhibitor (GABA receptor modulator)	0.4030	-0.6939

Pair number 14

Genbank	Description	Log2ratio Primary tumour	Log2ratio LNmeta
AA454868	platelet-derived growth factor receptor-like	-0.4319	0.3634
AA598601	insulin-like growth factor binding protein 3	-0.5738	1.3249
AA459100	tumor protein D52	-0.4685	0.4277
AA451904	epididymis-specific, whey-acidic protein type, putative ovarian carcinoma marker	-0.5549	0.4023
N53492	hypothetical protein FLJ20980	-0.3636	0.6295
R66101	neuritin	-1.0728	1.3768
AA455497	protein tyrosine phosphatase, receptor type, C	-0.4386	0.9715
AA457116	Homo sapiens cDNA FLJ14162 fis, clone NT2RM4002504	-0.4328	0.3119
AA136707	procollagen-lysine, 2-oxoglutarate 5-dioxygenase (lysine hydroxylase) 2	-0.6972	0.8675
W74079	CEGP1 protein	1.7371	-0.4615
AA485739	major histocompatibility complex, class II, DR beta 3	-0.5002	0.4047
W70234	dipeptidylpeptidase IV (CD26)	-0.8998	0.6627
AA461086	ESTs	-0.7132	0.5210
AA417363	KIAA0942 protein	-0.8465	0.4457
AA459980	protein kinase, cAMP-dependent, catalytic, beta	-0.7804	1.0808
N63575	KIAA0013 gene product	-0.5035	0.3190
AA775616	secreted phosphoprotein 1 (osteopontin, bone sialoprotein I)	-0.8565	1.7782
AA878048	keratin 15	0.8944	-0.6194
H23252	hypothetical protein FLJ20533	-1.1071	0.5182
AA664081	ESTs	-0.4635	0.4022
AA465697	BCL2/adenovirus E1B 19kD-interacting protein 3-like	-0.5804	0.4480
AA128200	coxsackie virus and adenovirus receptor	-0.7932	0.7899
AA191353	hypothetical protein FLJ23399	-0.4321	0.3853
AI417982	cartilage intermediate layer protein, nucleotide pyrophosphohydrolase	-0.9608	0.5634

Pair number 15

Genbank	Description	Log2ratio Primary tumour	Log2ratio LNmeta
AA447781	lumican	0.4064	-0.3513
H82536	cyclic nucleotide gated channel beta 1	0.8048	-0.4600
R31701	ESTs	0.7403	-0.5024
R75635	collagen, type V, alpha 1	0.7114	-0.4585
R00859	cathepsin K (pycnodysostosis)	0.5412	-0.6761
AA447761	aminolevulinate, delta-, synthase 1	0.7438	-0.4440
AA426227	uridine monophosphate synthetase	0.6138	-0.4476
AA490462	AE-binding protein 1	0.7661	-0.5477
AA598653	osteoblast specific factor 2 (fasciclin I-like)	1.1160	-1.8219
AA436142	sparc/osteonectin, cwcv and kazal-like domains proteoglycan (testican)	0.6814	-0.6811
AA478543	A kinase (PRKA) anchor protein (gravin) 12	0.7225	-0.5383
R71093	serine (or cysteine) proteinase inhibitor, clade H (hsp 47), member 2	0.7999	-0.8526

W72294	small inducible cytokine subfamily B (Cys-X-Cys), member 14 (BRAK)	-0.8817	1.0396
H95960	secreted protein, acidic, cysteine-rich (osteonectin)	0.7853	-0.3601
AA405569	fibroblast activation protein, alpha	1.0100	-0.4277
AA634109	Fc fragment of IgG, low affinity IIa, receptor for (CD32)	0.5026	-0.5598
N92901	fatty acid binding protein 4, adipocyte	0.5461	-0.6283
H08839	ESTs	-1.0768	0.5597
AA186427	TRAF interacting protein	0.6079	-1.0014
AA479883	hypothetical protein FLJ21127	1.1386	-0.5814
AA158211	hypothetical protein DKFZp434G0522	0.7607	-0.8671
AA449321	Homo sapiens cDNA FLJ12280 fis, clone MAMMA1001744	0.6367	-0.8764
AA777187	cysteine-rich, angiogenic inducer, 61 (IGFBP10)	0.8537	-0.5199
AA099554	a disintegrin and metalloproteinase domain 12 (meltrin alpha)	0.4135	-0.7937
AA775270	Homo sapiens mRNA; cDNA DKFZp586E2023	0.5572	-0.4019
AA410434	ESTs, Weakly similar to ORF YGL050w [S.cerevisiae]	0.5529	-0.5104
R48844	collagen, type I, alpha 1	0.8926	-1.1222
AA988574	vacuolar protein sorting 29 (yeast homolog)	0.4988	-0.6464
AA425227	matrix metalloproteinase 9 (gelatinase B, 92kD gelatinase,)	0.3291	-0.8679
AA450363	phosphatidylinositol glycan, class F	0.7539	-0.5092
AI341604	37 kDa leucine-rich repeat (LRR) protein	0.6733	-0.3899
AI356709	Melanoma associated gene	0.3445	-0.8710
AI262129	osteoblast specific factor 2 (fasciclin I-like)	0.9674	-1.6089
AA012944	cysteine-rich, angiogenic inducer, 61 (IGFBP10)	0.8228	-0.4934
H59614	insulin-like growth factor 2 (somatomedin A)	0.6102	-0.5620

Pair number 16

Genbank	Description	Log2ratio Primary tumour	Log2ratio LNmeta
AA039370	Homo sapiens transcribed sequence with similarity to protein pir:A40032	-0.7175	1.2496
AA158396	major histocompatibility complex, class II, DO beta	-0.3579	0.3742
AA011057	lectin, galactoside-binding, soluble, 7 (galectin 7)	0.4264	-0.6164
R69796	EST	-0.3719	0.7007
AA490462	AE-binding protein 1	0.7933	-0.3144
H08561	Human insulin-like growth factor binding protein 5 (IGFBP5) mRNA	0.7877	-1.0211
H62387	immunoglobulin superfamily containing leucine-rich repeat	0.9129	-0.3887
AA063521	BCL2/adenovirus E1B 19kD-interacting protein 3	-0.5322	1.4025
H22653	glia maturation factor, beta	-0.6857	0.6345
AA487429	ATP-binding cassette, sub-family B (MDR/TAP), member 2	0.6102	-0.3313
AA423957	thrombospondin 4	0.8583	-0.3184
AA521490	KIAA0430 gene product	0.3459	-0.3249
AA425947	dickkopf (Xenopus laevis) homolog 3	0.8800	-0.5657
AA436142	sparc/osteonectin, cwcv and kazal-like domains proteoglycan (testican)	0.3075	-0.6668
AA448157	cytochrome P450, subfamily I (dioxin-inducible), polypeptide 1	-0.3225	0.7498
AA449742	coagulation factor XIII, A1 polypeptide	1.0394	-0.7750
H58873	solute carrier family 2 (facilitated glucose transporter), member 1	-0.6623	0.4977
R05934	KRAB-zinc finger protein SZF1-1	-0.4882	0.7092
R00226	hypothetical protein FLJ21620	-0.6335	0.4490
W49799	neuronal protein	0.3585	-0.3911
H58645	tumor endothelial marker 8	0.6512	-0.3397
R78580	hypothetical protein from EUROIMAGE 1759349	1.2528	-0.4160

AA446839	BCL2/adenovirus E1B 19kD-interacting protein 3	-0.4271	1.5399
AA131664	hypothetical protein FLJ20277	0.7775	-0.9175
AA126982	sin3-associated polypeptide, 30kD	-0.3500	0.3884
N35241	Ser-Thr protein kinase related to the myotonic dystrophy protein kinase	0.4984	-1.3273
AA496149	3-hydroxy-3-methylglutaryl-Coenzyme A synthase 2 (mitochondrial)	-0.4445	2.2565
N93924	replication factor C (activator 1) 4 (37kD)	-0.6214	0.4627
R56123	ESTs, Weakly similar to fatty acid omega-hydroxylase [H.sapiens]	1.2220	-1.0693
AA485713	COBW-like protein	-0.7016	0.4691
AA669674	eukaryotic translation initiation factor 3, subunit 6 (48kD)	-0.3818	0.4788
AA449321	Homo sapiens cDNA FLJ12280 fis, clone MAMMA1001744	0.9366	-0.3829
AA478623	cathepsin B	0.6831	-0.4311
AA463639	eukaryotic translation elongation factor 1 alpha 1	-0.5679	0.5177
AA775616	secreted phosphoprotein 1 (osteopontin, bone sialoprotein I)	-0.3693	1.7519
H21977	ESTs, Weakly similar to CP4Y_HUMAN CYTOCHROME P450 4A11	2.1637	-0.9491
H98619	LCHN protein	-0.7112	0.6463
AA465147	SH3-containing protein SH3GLB1	-0.3612	0.5184
AA670296	translocase of inner mitochondrial membrane 10 (yeast) homolog	0.6668	-0.6178
AI005515	hexokinase 2	-0.8053	1.2195
R39162	DNA sequence from clone RP1-310O13 on chromosome 20q11.2.	0.7257	-0.7867
H05772	ESTs	-1.4952	0.3135
H17315	EST	-0.6447	0.7284
AA976544	hypothetical protein MGC2771	0.7978	-0.2988
AA479202	tissue inhibitor of metalloproteinase 3 (Sorsby fundus dystrophy)	0.6938	-0.8180
AA086038	phospholipase A2 receptor 1, 180kD	0.5338	-0.6645
AI359985	serine (or cysteine) proteinase inhibitor, clade A (alpha-1 antiproteinase, antitrypsin), member 8	0.4286	-0.4382
AI360356	synaptophysin	0.3660	-0.4977
AI365571	regulatory factor X-associated protein	0.4672	-0.8529
R37234	ankylosis, progressive (mouse) homolog	0.4291	-0.6195
AA417710	Homo sapiens mRNA; cDNA DKFZp761B1514	-0.3799	0.3721

Pair number 17

Genbank	Description	Log2ratio Primary tumour	Log2ratio LNmeta
H20822	Fc fragment of IgG, low affinity IIIb, receptor for (CD16)	-0.5264	0.4889
R38102	KIAA0203 gene product	-0.5253	0.6594
R41779	KIAA0156 gene product	-0.2861	0.4775
H08820	isopentenyl-diphosphate delta isomerase	-0.4369	0.4538
AA458653	GS3955 protein	-0.4813	1.3036
H24688	SWI/SNF related, matrix associated, actin dependent regulator of chromatin, subfamily c, member 2	-0.4445	0.5371
AA454852	proteasome (prosome, macropain) 26S subunit, non-ATPase, 2	0.5342	-0.3871
H22653	glia maturation factor, beta	-0.9055	0.5188
R91950	cytochrome b-5	0.3519	-0.5536
AA478589	apolipoprotein E	-0.5928	0.4726
H63077	annexin A1	-1.4213	0.4698
N93428	complement component 3	-0.6910	0.9757
AA399473	tissue factor pathway inhibitor 2	0.3987	-0.3873
AA278759	proteoglycan 1, secretory granule	-0.4632	0.4639
AA405562	protein phosphatase 4 (formerly X), catalytic subunit	-0.3753	0.4836

AA598795	Homo sapiens cDNA FLJ41727 fis, clone HLUNG2015578, highly similar to SERINE/THREONINE PROTEIN PHOSPHATASE 2A	-0.6199	0.4370
AA598517	keratin 8	0.3058	-0.5841
AA490996	interferon, gamma-inducible protein 16	-0.4786	0.4897
R65792	uncharacterized hypothalamus protein HCDASE	-0.5295	0.3965
T96603	hypothetical protein FLJ14153	-0.8563	0.4070
N91900	ESTs, Weakly similar to endo-alpha-D-mannosidase [R.norvegicus]	-0.4329	0.5514
R76553 | R76276	a disintegrin-like and metalloprotease (reprolysin type) with thrombospondin type 1 motif, 1	-0.5177	0.4657
N95358	Homo sapiens cDNA FLJ20153 fis, clone COL08656	-0.5207	0.7492
N77877	hypothetical protein DC42	-1.0916	0.4240
W69216	ESTs	-0.3501	0.4129
T70586	perilipin	-1.6744	0.7481
AA148641	Meis (mouse) homolog 2	-0.8851	0.5018
AA136707	procollagen-lysine, 2-oxoglutarate 5-dioxygenase (lysine hydroxylase) 2	-0.6361	0.5956
W90128	X-box binding protein 1	0.3382	-0.4193
AA496628	non-metastatic cells 2, protein (NM23B) expressed in	-0.5164	0.5834
AA633835	lipoprotein lipase	-0.8206	1.6735
AA056013	Microfibril-associated glycoprotein-2	-0.8290	0.4884
T56281	RNA helicase-related protein	-0.6660	0.4108
AA634109	Fc fragment of IgG, low affinity IIa, receptor for (CD32)	-1.7926	1.4330
N92901	fatty acid binding protein 4, adipocyte	-1.4594	1.5052
AA430367	cystathionine-beta-synthase	-0.7784	0.4764
AA401441	B-factor, properdin	0.3235	-0.8560
R55747	ESTs	-0.5203	0.5017
H17623	ESTs	-0.5749	0.5324
AA429946	peroxisomal short-chain alcohol dehydrogenase	0.5300	-0.4026
W86182	pinin, desmosome associated protein	-0.4580	0.6824
AA463512	CGI-99 protein	-0.4573	0.5855
AA452813	GK001 protein	-0.4682	0.6468
AA195021	GK001 protein	-0.3977	0.8468
AA406266	hypothetical protein FLJ23309	0.4526	-0.3255
AA775616	secreted phosphoprotein 1 (osteopontin, bone sialoprotein I)	-0.9310	1.4186
AA101875	chondroitin sulfate proteoglycan 2 (versican)	-0.3484	0.8963
AA857496	matrix metalloproteinase 10 (stromelysin 2)	-0.7263	0.7030
AA485214	nucleobindin 2	-0.7848	0.6678
H19804	ESTs	-0.5458	0.3369
AA291972	ubiquinol-cytochrome c reductase (6.4kD) subunit	0.7573	-0.3753
AA775840	Homo sapiens mRNA; cDNA DKFZp434O1230	0.9209	-0.3676
H23252	hypothetical protein FLJ20533	0.9098	-1.2176
AA010211	ESTs	-0.4816	1.0804
AA968514	WW domain binding protein 1	0.4379	-0.4273
AA888213	DnaJ (Hsp40) homolog, subfamily C, member 8	-0.6011	0.5191
AA521388	RuvB (E coli homolog)-like 1	0.3141	-0.3799
AI352345	carbonyl reductase 3	0.6860	-0.6194
AA878527	kinectin 1 (kinesin receptor)	-0.6879	0.5259
AI206454	fatty-acid-Coenzyme A ligase, long-chain 3	-0.6383	0.3319
AI420743	alcohol dehydrogenase 3 (class I), gamma polypeptide	-0.9081	0.9956
AI653116	KIAA1077 protein	-1.0453	0.3592
