# Supplementary material for: No common denominator for breast cancer lymph node metastasis
Source: Br J Cancer. 2005 Sep 27;93(8):924–32. doi: 10.1038/sj.bjc.6602794 (PMC2361648; doi:10.1038/sj.bjc.6602794)
Supplement: Supplementary Table S3 [file 93-6602794x3.pdf]

Supplementary Table S3 Significant genes (p<0.01) anti-expressed between individual pairs of primary tumours and matching lymph node metastases, grouped by molecular function.

Pair number	Number of anti-expressed genes	Number of genes per functional group	Percentage
**1**	11	3 ECM/cell-matrix interaction (CD36, MMP3, MMP9)	27%
		2 immune response (serum amyloid A4, Fc of IgG)	18%
		2 ESTs/hypothetical proteins	18%
		other	37%
**3**	32	2 ECM/cell-matrix interaction (TIMP-3, chitinase 3-like 1)	6.25%
		2 growth factor (t-PA, GDNF family receptor alpha 1)	6.25%
		2 signaling (cAMP-dependent protein kinase inhibitor beta/PKI inhibitor, MAP kinase phosphatase 6)	6.25%
		1 cell cycle (MRE11A)	3.13%
		5 immune response (FC of IgG, serum amyloid A4, IL1receptor, interferon induced protein, IL enhancer binding factor)	15.63%
		6 ESTs/hypothetical proteins	18.75%
		other	43.74%
**4**	3	2 ECM/cell-matrix interaction (osteopontin, hypothetical protein FLJ20277 anchor basement membranes to the underlying connective tissue)	67%
		1 EST	33%
**5**	9	2 ECM/cell-matrix interaction (MMP11, Microfibril-associated glycoprotein-2)	22.20%
		1 growth factor (IGFBP3)	11.10%
		1 immune response (hypothetical protein FLJ22418 T cell costimulatory molecule B7x)	11.10%
		2 ESTs/hypothetical proteins	22.20%
		other	33.30%
**6**	129	13 ECM/cell-matrix interaction (MMP1, MMP7, Integrin beta 1, mucin 1, CD44, osteopontin, VCAM-1, capping protein (actin filament) alpha 1,	10.10%
		actin related protein 2/3 complex, ESTs R63694 (proteoglycan 4), cDNA DKFZp434E2023 (COL14A1), ArgBP2, delta-like homolog)	
		1 growth factor (IGFBP3)	0.78%
		2 cell cycle (CDC5, Fanconi anemia complementation group G)	1.60%
		8 signaling (protein tyrosine phosphatase RC, RAP1A, MEKK5, 2x frizzled related protein, p55 gamma, RBP1-like protein, DARPP-32)	6.20%
		11 immune (FC of IgG, MHC, T-cell proliferation, IF gamma receptor, IF gamma inducible protein, Ig (CD79A) binding protein 1, lacto-	8.50%
		transferrin, SB classII histocompatibility antigen alpha-chain, S100 calcium-binding protein A9, serum amyloid A4, pentaxin-related gene)	
		26 ESTs/hypothetical proteins	20.20%
		other	52.62%

Pair number	Number of anti-expressed genes	Number of genes per functional group	Percentage
**7**	19	2 ECM/cell-matrix interaction (MMP3, ARP1)	10.53%
		2 immune response (serum amyloid, killer cell lectin-like receptor subfamily C)	10.53%
		7 ESTs/hypothetical proteins	36.80%
		other	42.14%
**8**	35	3 ECM/cell-matrix interaction (2x wingless-type MMTV integration site family member 2, L Selectin)	8.60%
		2 signaling (WW Domain-Containing Gene, bradykinin receptor B2)	5.70%
		13 immune response (complement component 7, CD79A antigen, immunoglobulin lambda locus, phospholipase A2, preferentially	37%
		expressed antigen in melanoma, immunoglobulin lambda-like polypeptide, immunoglobulin heavy constant mu, 2x immunoglobulin, kappa	
		variable 3D-15, T cell receptor beta chain, interferon-stimulated protein, immunoglobulin lambda light chain variable region, G antigen 5)	
		3 ESTs/hypothetical proteins	8.60%
		other	40%
**9**	20	3 ECM/cell-matrix interaction (collagen XVII, ESTs AA399633 (involved in cell-cell interactions, carboxypeptidase X2), Ig superfamily protein)	15%
		4 growth factor (IGF2, IGFBP10, IGF1, IGFBP10)	20%
		1 cell cycle (MRE11A)	5%
		1 signaling (ESTs W73366 (recognizes activated receptor tyrosine kinases, including PDGFA, EGF, CSF1, signaling))	5%
		2 immune response (Lymphotoxin beta, T-cell leukemia)	10%
		5 ESTs/hypothetical proteins	25%
		other	20%
**10**	19	2 ECM/cell-matrix interaction (sarcoglycan epsilon, syndecan 2)	10.50%
		1 growth factor (DKFZP564I1922 protein (VEGFR activity))	5.30%
		1 cell cycle (cyclin G associated kinase)	5.30%
		5 immune response (2x major histocompatibility complex, immunoglobulin kappa variable 3D-15, prominin (mouse)-like 1, lymphotoxin beta)	26.30%
		4 ESTs/hypothetical proteins	21.10%
		other	21.50%
**11**	23	4 ECM/cell-matrix interaction (2x discs, 2x matrix Gla protein)	17.40%
		4 signaling (protein kinase H11, signal transduction protein, signal transducer + activator of transcription 4, glycoprotein hormones)	17.40%
		2 cell cycle (cell cycle protein 2 protein, p27)	8.70%
		2 immune (2x major histocompatibility complex)	8.70%
		5 ESTs/hypothetical proteins	21.70%
		other	26.10%

Pair number	Number of anti-expressed genes	Number of genes per functional group	Percentage
**12**	149	7 ECM/cell-matrix interaction (MMP11, integrin alpha 2, CD44, matrix Gla protein, COL2A1, fibulin 1, MARCKS 80K-L)	4.70%
		1 growth factor (growth factor receptor-bound protein 7)	0.70%
		13 signaling (carcinoembryonic antigen-related cell adhesion molecule 6, v-erb-b2, IQ motif containing GTPase activating protein 2,	8.72%
		src homology three (SH3) and cysteine rich domain, Homo sapiens, clone MGC (Rho/rac guanine nucleotide exchange factor 2),	
		prolactin receptor, Norrie disease, kynureninase, bradykinin receptor B2, phosphoinositide-3-kinase, PRAME, GABA A receptor alpha 1,	
		ESTs (CAPNS2))	
		25 immune response (14x major histocompatibility complex, 3x complement factor, guanylate binding protein 2, immunoglobulin lambda	16.80%
		locus, CD164 antigen, immunoglobulin superfamily member 3, immunoglobulin heavy constant mu, EST (interferon induced transmembrane	
		protein 3), SerpinA3, tryptase beta 1)	
		3 apoptosis (caspase 1, pleckstrin homology-like domain family A member 1, Homo sapiens cDNA (PHLDA1))	2.01%
		26 ESTs/hypothetical proteins	17.50%
		other	49.57%
**14**	24	2 ECM/cell-matrix interaction (osteopontin, procollagen-lysine)	8.33%
		2 growth factor (platelet-derived growth factor receptor-like, insulin-like growth factor binding protein 3)	8.33%
		3 signaling (protein tyrosine phosphatase receptor type C, PKA C-beta, nucleotide pyrophosphohydrolase)	12.50%
		3 immune response (major histocompatibility complex, dipeptidylpeptidase IV (CD26), coxsackie virus and adenovirus receptor)	12.50%
		1 apoptosis (BCL2/adenovirus E1B 19kD-interacting protein 3-like)	4.20%
		6 ESTs/hypothetical proteins	25%
		other	29.14%
**15**	35	13 ECM/cell-matrix interaction (lumican, COL5A1, cathepsin K, Spock, osteoblast specific factor 2, MMP9, osteoblast specific factor 2,	37.10%
		COL1A1, COL5A1, ADAM12, osteonectin, fibroblast activation protein, AA410434 (ESTs, collagen triple helix repeat containing 1))	
		3 growth factor (2x cysteine-rich angiogenic inducer 61 (IGFBP10), IGF2)	8.60%
		1 signaling (TRAF interacting protein)	2.90%
		1 immune response (Fc fragment of IgG)	2.90%
		6 ESTs/hypothetical proteins	17.10%
		other	31.40%

Pair number	Number of anti-expressed genes	Number of genes per functional group	Percentage
**16**	51	6 ECM/cell-matrix interaction (galectin 7, thrombospondin 4, Spock, osteopontin, TIMP-3, cathepsin B)	11.80%
		1 growth factor (IGFBP5)	2.00%
		2 signaling (glia maturation factor beta, Ser-Thr protein kinase related to the myotonic dystrophy protein kinase)	3.90%
		1 cell cycle (hexokinase 2)	2.00%
		4 apoptosis (2x BCL2/adenovirus E1B 19kD-interacting protein 3,hypothetical protein FLJ21620 (EGLN3), SH3GLB1)	7.80%
		2 immune response (major histocompatibility complex, immunoglobulin superfamily containing leucine-rich repeat)	3.90%
		7 ESTs/hypothetical proteins	13.70%
		other	54.90%
**17**	62	8 ECM/cell-matrix interaction (tissue factor pathway inhibitor 2, proteoglycan 1, a disintegrin-like and metalloprotease with thrombospondin	12.90%
		type 1, MMP10, procollagen-lysine, osteopontin, chondroitin sulfate proteoglycan 2 (versican), Microfibril-associated glycoprotein-2)	
		5 signaling (glia maturation factor beta, annexin A, protein phosphatase 4, hypothetical protein IRAK-1, WW domain binding protein 1)	8.10%
		6 immune response (2x Fc fragment of IgG, complement component 3, interferon gamma-inducible protein 16, B-factor, properdin)	9.70%
		11 ESTs/hypothetical proteins	17.70%
		other	51.60%
